# Antibiotic Susceptibility of Clinical Burkholderia pseudomallei Isolates in Northeast Thailand from 2015 to 2018 and the Genomic Characterization of *β*-Lactam-Resistant Isolates

**DOI:** 10.1128/AAC.02230-20

**Published:** 2021-04-19

**Authors:** Shirley Yi Fen Hii, Sarunporn Tandhavanant, Rungnapa Phunpang, Peeraya Ekchariyawat, Natnaree Saiprom, Claire Chewapreecha, Rathanin Seng, Ekkachai Thiansukhon, Chumpol Morakot, Narongchai Sangsa, Sunee Chayangsu, Somchai Chuananont, Kittisak Tanwisaid, Wirayut Silakun, Noppol Buasi, Seksan Chaisuksant, Tanin Hompleum, Ploenchan Chetchotisakd, Nicholas P. J. Day, Wasun Chantratita, Ganjana Lertmemongkolchai, T. Eoin West, Narisara Chantratita

**Affiliations:** a Department of Microbiology and Immunology, Faculty of Tropical Medicine, Mahidol University, Bangkok, Thailand; b Mahidol-Oxford Tropical Medicine Research Unit, Faculty of Tropical Medicine, Mahidol University, Bangkok, Thailand; c Department of Microbiology, Faculty of Public Health, Mahidol University, Bangkok, Thailand; d Wellcome Sanger Institute, Hinxton, United Kingdom; e Bioinformatics and Systems Biology Program, School of Bioresource and Technology, King Mongkut’s University of Technology Thonburi, Bangkok, Thailand; f Department of Medicine, Udon Thani Hospital, Udon Thani, Thailand; g Department of Medicine, Mukdahan Hospital, Mukdahan, Thailand; h Department of Medicine, Roi Et Hospital, Roi Et, Thailand; i Department of Medicine, Surin Hospital, Surin, Thailand; j Department of Medicine, Nakhon Phanom Hospital, Nakhon Phanom, Thailand; k Department of Medicine, Buriram Hospital, Buriram, Thailand; l Department of Medicine, Sisaket Hospital, Sisaket, Thailand; m Department of Medicine, Khon Kaen Hospital, Khon Kaen, Thailand; n Department of Surgery, Khon Kaen Hospital, Khon Kaen, Thailand; o Department of Medicine, Srinagarind Hospital, Faculty of Medicine and Research and Diagnostic Center for Emerging Infectious Diseases (RCEID), Khon Kaen University, Khon Kaen, Thailand; p Center for Tropical Medicine and Global Health, University of Oxford, United Kingdom; q Center for Medical Genomics, Faculty of Medicine, Ramathibodi Hospital, Bangkok, Thailand; r Department of Clinical Immunology, Faculty of Associated Medical Science, Khon Kaen University, Khon Kaen, Thailand; s The Centre for Research and Development of Medical Diagnostic Laboratories, Khon Kaen University, Khon Kaen, Thailand; t Division of Pulmonary, Critical Care & Sleep Medicine, Harborview Medical Center, University of Washington, Seattle, Washington, USA

**Keywords:** β-lactam resistance, *penA*, melioidosis, genome, *Burkholderia pseudomallei*, ceftazidime, Thailand

## Abstract

Melioidosis is an often fatal infection in tropical regions caused by an environmental bacterium, Burkholderia pseudomallei. Current recommended melioidosis treatment requires intravenous β-lactam antibiotics such as ceftazidime (CAZ), meropenem (MEM), or amoxicillin-clavulanic acid (AMC) and oral trimethoprim-sulfamethoxazole. Emerging antibiotic resistance could lead to therapy failure and high mortality. We performed a prospective multicenter study in northeast Thailand from 2015 to 2018 to evaluate antibiotic susceptibility and characterize β-lactam resistance in clinical B. pseudomallei isolates. A collection of 1,317 B. pseudomallei isolates from patients with primary and relapse infections were evaluated for susceptibility to CAZ, imipenem (IPM), MEM, and AMC. β-Lactam-resistant isolates were confirmed by the broth microdilution method and characterized by whole-genome sequence analysis, *penA* expression, and β-lactamase activity. The resistant phenotype was verified via *penA* mutagenesis. All primary isolates were IPM susceptible, but we observed two CAZ-resistant isolates and one CAZ-intermediate isolate, two MEM-less-susceptible isolates, and one AMC-resistant and two AMC-intermediate isolates. One of 13 relapse isolates was resistant to both CAZ and AMC. Two isolates were MEM less susceptible. Strains DR10212A (primary) and DR50054E (relapse) were multidrug resistant. Genomic and mutagenesis analyses supplemented with gene expression and β-lactamase analyses demonstrated that the CAZ-resistant phenotype was caused by PenA variants: P167S (*n* = 2) and *penA* amplification (*n* = 1). Despite the high mortality rate in melioidosis, our study revealed that B. pseudomallei isolates had a low frequency of β-lactam resistance caused by *penA* alterations. Clinical data suggest that resistant variants may emerge in patients during antibiotic therapy and may be associated with a poor response to treatment.

## Abstract (precis)

Melioidosis is an often fatal infection in tropical regions caused by an environmental bacterium, Burkholderia pseudomallei. Current recommended melioidosis treatment requires intravenous β-lactam antibiotics such as ceftazidime (CAZ), meropenem (MEM), or amoxicillin-clavulanic acid (AMC) and oral trimethoprim-sulfamethoxazole.

## INTRODUCTION

Burkholderia pseudomallei is the causative agent for melioidosis, an often fatal disease with a predicted global burden of 165,000 cases per year and 89,000 deaths worldwide (1). Regions of melioidosis endemicity, including Southeast Asia and northern Australia, account for up to 40% (2) and 10% (3) case fatality rates, respectively. Transmission routes include percutaneous inoculation, inhalation, and ingestion of contaminated soil and water (4). The most common clinical presentations include pneumonia and bacteremia (40 to 60%) (5). B. pseudomallei is categorized as a Tier 1 Select Agent by the Centers of Disease Control and Prevention (CDC) (6). To date, there is no commercially available vaccine for melioidosis.

B. pseudomallei is intrinsically resistant to many antibiotics, including penicillin, ampicillin, and first- and second-generation cephalosporins (5). The recommended melioidosis treatment is biphasic therapy: an intensive phase of at least 10 to 14 days of intravenous (i.v.) β-lactam antibiotics, ceftazidime (CAZ) or meropenem (MEM), followed by an eradication phase of 3 to 6 months of oral trimethoprim-sulfamethoxazole (SXT) to eliminate residual bacteria (https://www.cdc.gov/melioidosis/treatment/index.html). Amoxicillin-clavulanic acid (AMC), a combination of a β-lactam and a β-lactamase inhibitor, is used as an alternative in both acute and eradication therapy (7). SXT, which has excellent tissue penetration, is also added to intensive-phase treatment for neurological involvement and deep-seated abscess (7).

CAZ alone is the first-line drug against melioidosis in Thailand due to its efficacy and lower cost in comparison to carbapenems. In Australia, MEM is preferred for severe melioidosis cases and is switched back to CAZ upon patients reaching stable conditions (5). Prolonged acute-phase therapy and oral SXT are required because these regimens have been associated with lower risk for relapse (8–11). Given the intrinsic antimicrobial resistance of the pathogen and few other therapeutic alternatives, emerging resistance against the limited drugs could be fatal. Primary CAZ resistance, although rare, has been reported at 0.1 to 1.5% in Thailand (12–14) and 0.6 to 2.4% in Malaysia (15–17). Acquired resistance has been observed in patients who received prolonged or multiple courses of CAZ (13, 18–23). The currently known mechanisms conferring CAZ resistance in B. pseudomallei have been described in some studies. For examples, alterations of the membrane-bound class A β-lactamase gene *penA* (*BPSS0946*; current NCBI locus tag, *BPS_RS23870*) include the following mechanisms: (i) elevated *penA* expression due to promoter mutation (−78G>A) (23–25); (ii) gene duplication and amplification (GDA) event in the *penA* chromosomal region (21, 26); and (iii) *penA* point mutation at C69Y (18, 21, 23, 27), P167S (20, 22, 27), and D240G (25). Our previous study revealed several CAZ-resistant isolates in Thai patients caused by deletion of the β-lactam target, penicillin-binding protein 3 (PBP3) (*BPSS1219*; current NCBI locus tag, *BPS_RS25365*) (19). Elevated expression of the class D β-lactamase gene *oxa* was also observed in laboratory-generated CAZ-induced resistant mutants (28, 29). In addition, an amino acid change in PenA involving S72F (22, 25, 27) and T147A (25) was reported to confer resistance toward AMC or both AMC and IPM, respectively. Alterations in B. pseudomallei-encoded resistance nodulation and cell division (RND) multidrug efflux pumps AmrAB-OprA, BpeAB-OprB, and BpeEF-OprC have been implicated in antibiotic resistance. For instance, isolates harboring mutations affecting AmrR, BpeR, BpeT, and BpeS increased the MIC of MEM and SXT (21, 24, 30–32).

In this study, we aimed to evaluate the performance of β-lactam antibiotics in melioidosis treatment *in vitro*. Antibiotic susceptibility testing was conducted on 1,317 clinical B. pseudomallei isolates from 1,304 patients in nine hospitals across northeast Thailand from 2015 to 2018. We used whole-genome sequencing (WGS) to investigate genotypes and to search for putative mutations in β-lactam-resistant B. pseudomallei strains. Antibiotic treatment of these patients was investigated. Genomic and functional characterizations of *penA* were evaluated via reverse transcription real-time PCR (RT-PCR), β-lactamase activity, and mutagenesis.

## RESULTS

### Initial screening of antibiotic susceptibility in B. pseudomallei isolates.

**FIG 1:**
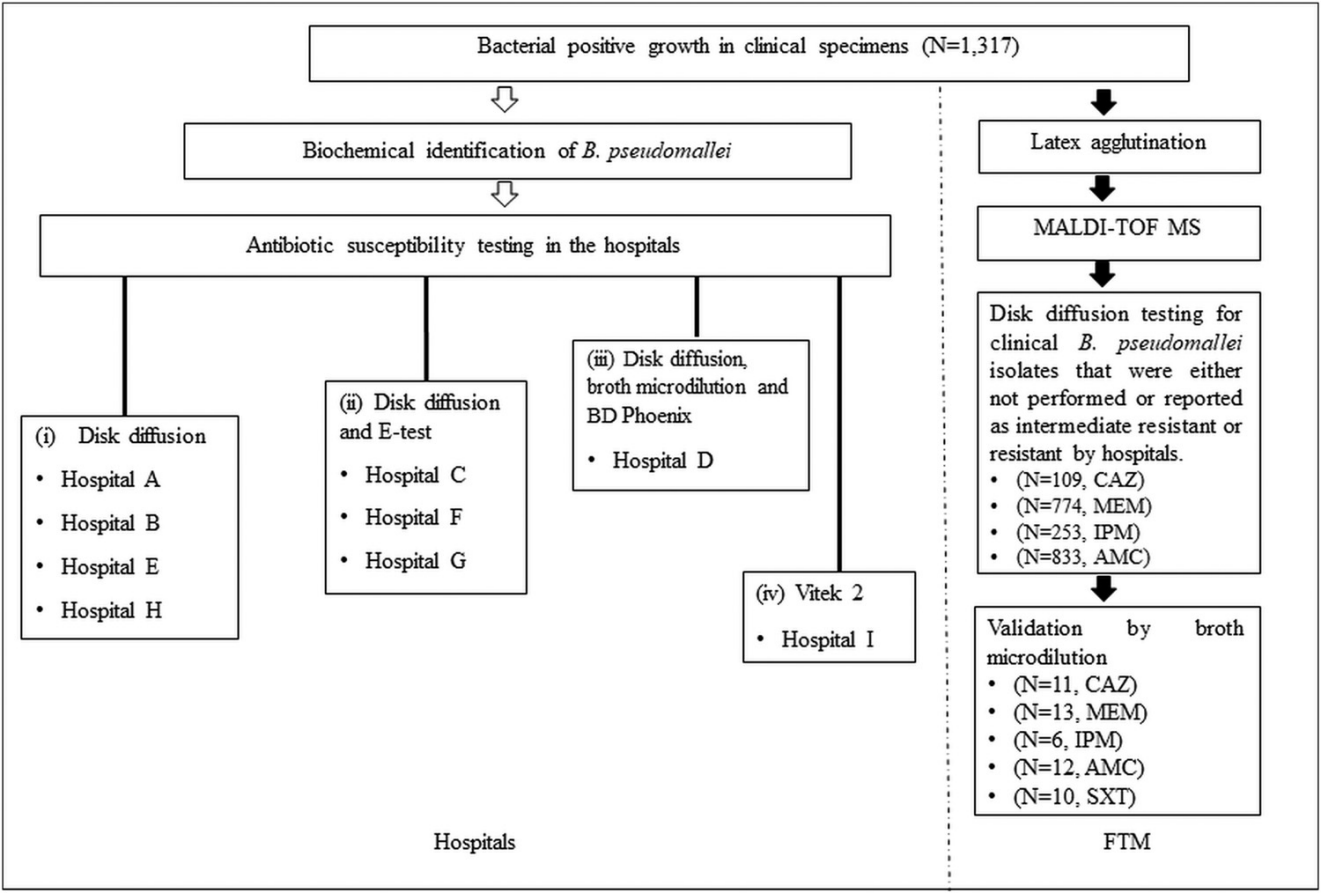
Flow chart for identification and antibiotic susceptibility testing of Burkholderia pseudomallei at nine hospitals in northeast Thailand and at the Faculty of Tropical Medicine, Mahidol University (FTM).

Initial screening of antibiotic susceptibility for 1,317 B. pseudomallei isolates was performed at nine hospitals (Table 1), and subsequent evaluation was performed at the Faculty of Tropical Medicine, Mahidol University (FTM), by methods as described in Fig. 1. Six hospitals reported resistant and intermediate isolates for CAZ, AMC, MEM, and IPM: hospital A (*n* = 3), hospital C (*n* = 1), hospital D (*n* = 1), hospital E (*n* = 1), hospital H (*n* = 4), and hospital I (*n* = 8) (Table 2). In hospital A, one isolate, DR10212A, was both CAZ resistant (CAZ-R) and MEM resistant (MEM-R) and two isolates were AMC intermediate (AMC-I). Hospital D reported one ceftazidime-intermediate (CAZ-I) isolate. Hospital H reported two MEM-R isolates, and FTM found one isolate each of AMC-R and AMC-I. For hospital I, upon checking on hospital laboratory reports, we observed that the Vitek 2 advanced expert system (AES) interpreted one isolate as CAZ-R, MEM-R, and IPM resistant (IPM-R), one as MEM intermediate (MEM-I), and five isolates as IPM-R. FTM observed one additional MEM-I isolate by disk diffusion testing (DD). We also found one isolate reported as CAZ-R at hospital C and one isolate with both CAZ-R and AMC-R at hospital E.

**TABLE 1:** Number of Burkholderia pseudomallei isolates and clinical specimens from melioidosis patients at nine hospitals in northeast Thailand

Hospital designation	Total no. of isolates	No. of isolates from indicated type of clinical specimen						
		Blood	Respiratory secretion	Body fluid^*a*^	Pus^*b*^	Urine	Wound swab	Tissue^*c*^
A	227	185	11	16	14	0	1	0
B	89	74	9	1	5	0	0	0
C	26	12	0	3	7	0	1	3
D	140	96	20	3	18	3	0	0
E	226	149	30	5	35	7	0	0
F	198	135	26	6	23	6	0	2
G	127	86	10	3	23	5	0	0
H	177	128	16	5	23	3	1	1
I	107	75	11	2	16	2	0	1
Total	1,317	940	133	44	164	26	3	7

a Body fluids from peritoneum, cerebrospinal, pericardium, pleural cavity, liver, synovial joints, elbow, joint, ankle, knee, or bile.

b Pus from liver, kidney, neck, shoulder, arm, leg, ankle, knee, or foot.

c Tissue from aneurysmal wall, lymph node, or unknown origin.

**TABLE 2:** Antibiotic susceptibility interpretation of initial screening and validation of nonsusceptible B. pseudomallei isolates

Hospital and B. pseudomallei isolate^*a*^	Isolate	Clinical specimen	Antibiotic^*b*^	Susceptibility interpretation^*c*^		
				Initial screen^*d*^		Validation at FTM^*e*^ (BMD)
				Hospital	FTM (DD)	
Hospital A
DR10212A	Primary	Pleural fluid	CAZ	**R**	**R**	**R**
			MEM	**R**	**R**	**LS**
DR10118A	Primary	Blood	AMC	**I**	S	S
DR10120A	Primary	Blood	AMC	**I**	S	S
Hospital C
DR30013A	Primary	Blood	CAZ	NA	**R**	**R**
Hospital D
DR40031A	Primary	Blood	CAZ	**I**	S	S
AMC	ND	S	**I**
Hospital E
DR50054E	Relapse	Sputum	AMC	ND	**R**	**R**
CAZ	S	**R**	**R**
MEM	S	S	**LS**
Hospital H
DR80176A	Primary	Sputum	MEM	**R**	S	S
DR80177A	Primary	Blood	MEM	**R**	S	S
DR80109A	Primary	Sputum	AMC	ND	**I**	**I**
DR80110A	Primary	Urine	AMC	ND	**R**	**R**
CAZ	S	S	**I**
Hospital I
DR90087A	Primary	Blood	CAZ	**R**	S	S
	MEM	**R**	S	S
	IPM	**R**	S	S
DR90049A	Primary	Blood	MEM	**I**	S	**LS**
DR90003A	Primary	Blood	IPM	**R**	S	S
DR90026A	Primary	Sputum	IPM	**R**	S	S
DR90036A	Primary	Blood	IPM	**R**	S	S
DR90045A	Primary	Sputum	IPM	**R**	S	S
DR90076A	Primary	Body fluid	IPM	**R**	S	S
DR90031E	Relapse	Pus	MEM	NA	**I**	**LS**

a A, primary isolate; E, relapse isolate.

b CAZ, ceftazidime; IPM, imipenem; MEM, meropenem; AMC, amoxicillin-clavulanic acid.

c S, susceptible; I, intermediate; R, resistant; LS, less susceptible for meropenem; ND, not done; NA, not available; FTM, Faculty of Tropical Medicine, Mahidol University; DD, disk diffusion test.

d Antibiotic susceptibility testing screening by hospitals and FTM as described in Fig. 1.

e Validation by broth microdilution method (BMD) by FTM. Boldface indicates antibiotic-resistant or intermediate isolates.

### Validation of antibiotic susceptibility of B. pseudomallei isolates.

Broth microdilution dilution (BMD) assessment was used to validate the antibiotic nonsusceptibility and discrepant results of initial screenings between hospitals and FTM for CAZ, MEM, IPM, AMC, and SXT. Six isolates from hospital I originally reported to be IPM-R were found at FTM to have MICs within the susceptible range (Table 2).

Three clinical isolates were confirmed as CAZ-R (strains DR10212A, DR30013A, and DR50054E [primary and relapse isolates are denoted by “A” and “E,” respectively, at the end of the strain name]) with MICs of 128, 64, and 64 μg/ml, respectively, and one isolate (DR80110A) that was initially determined to be CAZ-S was reinterpreted as CAZ-I with an MIC of 16 μg/ml (Table 3). DR10212A, DR30013A, and DR80110A were defined as primary isolates. We observed that the relapse isolate DR50054E had an increased MIC of CAZ of 64 μg/ml compared to the first-episode isolate DR50054A, which exhibited an MIC of only 2 μg/ml.

**TABLE 3:** Antibiotic susceptibility profile of B. pseudomallei isolates analyzed in this study and CLSI susceptibility breakpoints for antibiotics^*a*^

Strain or interpretation	Isolate	MIC (μg/ml)			
		CAZ	MEM	AMC	SXT
K96243	Laboratory	4 (S)	1 (S)	8/4 (S)	2/38 (S)
DR10212A	Primary	128 (**R**)	8 (**LS**)	8/4 (S)	4/76 (**R**)
DR30013A	Primary	64 (**R**)	1 (S)	2/1 (S)	2/38 (S)
DR80110A	Primary	16 (**I**)	2 (S)	32/16 (**R**)	0.5/9.5 (S)
DR90049A	Primary	2 (S)	4 (**LS**)	8/4 (S)	0.5/9.5 (S)
DR40031A	Primary	8 (S)	1 (S)	16/8 (**I**)	1/19 (S)
DR80109A	Primary	1 (S)	1 (S)	16/8 (**I**)	0.5/9.5 (S)
DR50054A	Primary	2 (S)	1 (S)	8/4 (S)	1/19 (S)
DR50054E	Relapse	64 (**R**)	4 (**LS**)	32/16 (**R**)	0.5/9.5 (S)
DR90031A	Primary	2 (S)	1 (S)	4/2 (S)	0.25/4.75 (S)
DR90031E	Relapse	2 (S)	4 (**LS**)	4/2 (S)	0.25/4.75 (S)
CLSI susceptibility breakpoint interpretation (μg/ml)	S	≤8	NA	≤8/4	≤2/38
	I	16	NA	16/8	NA
	R	≥32	NA	≥32/16	≥4/76

a MICs for each isolate were determined after 18 h of incubation at 37°C and interpretation as recommended in Clinical Laboratory and Standards Institute (CLSI) M45, 2016 (39). Interpretation for MEM is not available for B. pseudomallei in CLSI. In this study, MEM-LS refers to isolates with an MIC of >2 μg/ml (47). CAZ, ceftazidime; MEM, meropenem; AMC, amoxicillin-clavulanic acid; SXT, trimethoprim-sulfamethoxazole; R, resistant; LS, less susceptible; I, intermediate; S, susceptible; NA, not available. Boldface indicates antibiotic-resistant or intermediate isolates.

The BMD results for MEM confirmed four isolates as MEM less susceptible (MEM-LS) with MICs of 4 μg/ml (DR90049A, DR50054E, and DR90031E) and 8 μg/ml (DR10212A) (Table 3). The MICs of two relapse isolates, DR50054E and DR90031E, were both 4-fold higher than those of their primary pair isolates, DR50054A and DR90031A (4 μg/ml versus 1 μg/ml for both pairs).

The BMD results for AMC confirmed two AMC-R isolates with MICs of 32/16 μg/ml (DR80110A and DR50054E) and two AMC-I isolates (DR40031A and DR80109A) with MICs of 16/8 μg/ml (Table 3). For the relapse pair isolates, only DR50054E was AMC-R, with an increased MIC of 32/16 μg/ml compared to an MIC of 8/4 μg/ml for the primary isolate, DR50054A.

We also tested susceptibility to SXT, which is used as a treatment option in some patients who are not responsive to β-lactams. Using BMD, we observed that DR10212A was SXT resistant (SXT-R) with an MIC of 4/76 μg/ml. In contrast, DR30013A, DR80110A, DR90049A, DR40031A, DR80109A, as well as relapse pair isolates DR50054A, DR50054E, DR90031A, and DR90031E were all SXT susceptible (SXT-S) (Table 3).

### Prevalence of antibiotic-resistant B. pseudomallei in northeast Thailand.

To determine the prevalence of antibiotic resistance or intermediate resistance to clinically relevant drugs used in treatment against B. pseudomallei, a combination of DD and BMD was used to interpret the results (Tables 2 and 3). Decreased susceptibility to one or more β-lactam antibiotics tested was observed for six primary isolates (6/1,304, 0.46%), consisting of CAZ-R (*n* = 2), CAZ-I (*n* = 1), MEM-LS (*n* = 2), AMC-R (*n* = 1), and AMC-I (*n* = 2). These included one isolate (DR10212A) exhibiting both CAZ-R and MEM-LS (1/227, 0.44%) in hospital A, one CAZ-R (1/26, 3.85%) isolate (DR30013A) in hospital C, and one AMC-I (1/140, 0.71%) isolate (DR40031A) in hospital D. In hospital H, of two isolates with reduced antibiotic susceptibility, one isolate (DR80110A) was AMC-R and CAZ-I (1/177, 0.57%) and another isolate (DR80109A) was AMC-I. One MEM-LS (1/107, 0.94%) primary isolate (DR90049A) was observed in hospital I.

For relapse isolates, 2/13 (15.4%) had developed resistance to previously completed antibiotics. One relapse isolate (DR50054E) from hospital E was CAZ-R, MEM-LS, and AMC-R. The other relapse isolate (DR90031E) collected from hospital I was MEM-LS.

Taken together, two multidrug-resistant (MDR) isolates, DR10212A (CAZ-R, MEM-LS, SXT-R) and DR50054E (CAZ-R, MEM-LS, AMC-R) were observed from this cohort.

### Treatment history of patients infected with B. pseudomallei with decreased susceptibility to antibiotics.

We evaluated the medical records of six patients who were infected with B. pseudomallei with decreased susceptibility to CAZ or MEM for clues to the development of antibiotic resistance (Table 4). Four patients were transferred from other hospitals to the study hospital sites, so the initial treatment histories were incomplete. The overall mortality of melioidosis patients in this cohort was 33%. Two of six (33%) patients with CAZ- or MEM-nonsusceptible isolates eventually died at day 40 (patient 1) and day 9 (patient 2) after admission. They had received CAZ or MEM for at least 9 days prior to the isolation of B. pseudomallei with less susceptibility to the administered antibiotics. Patient 4 was admitted upon positive culture for B. pseudomallei (AMC-R) and had received an unknown duration of AMC medication from the referring hospital. A positive B. pseudomallei (MEM-LS) isolate was cultured from a blood specimen from patient 6. The patient had a history of melioidosis 2 years earlier and was not treated with MEM in the current admission. For the other two relapse cases, only patient 3 had received CAZ, while patient 5 had been treated with AMC instead of CAZ or MEM during the initial treatment course.

**TABLE 4:** Details of six patients with B. pseudomallei isolates resistant or less susceptible to β-lactam antibiotics*^a^*

Patient no. (study hospital)	Clinical presentation and medical history	Day and type of specimen collection after admission	Isolate ID (antibiotic susceptibility result)	Treatment received after admission in study hospital			Patient status
				Antibiotic	Start (day)	End (day)	
1* (hospital A)	Fever, underlying diabetes mellitus, history of left lobectomy, required mechanical ventilation, empyema thoracis, and bronchopleural fistula (consecutive drainages done on days 8, 9, 23, and 36); coinfected with MDR A. baumannii; patient was provisionally treated as melioidosis and had received an unknown duration of CAZ in a referral hospital	9 (pleural fluid)	DR10212A (CAZ-R, MEM-LS, SXT-R)	MEM	0	13	Died at day 40
				MEM	21	39	
				AMC	13	22	
2* (hospital C)	Poorly controlled type 2 diabetes mellitus and fever; patient had received CTR (3 days) and CAZ (9 days) from a referral hospital	0 (blood)	DR30013A (CAZ-R)	CAZ	0	7	Died at day 9
				SXT	4	9	
				CST	4	5	
				MEM	7	9	
				VAN	5	6	
				VAN	8	9	
3 (hospital E)	Underlying TB with ongoing treatment, hemoptysis, chronic obstructive lung disease, and renal impairment	1 (sputum)	DR50054A (S)	CAZ	0	16	Discharged at day 22 with home oral AMC
				AZM	0	4	
				STR	1	21	
				SXT	12	19	
Relapsed infection	354 days after first episode (sputum)	DR50054E (CAZ-R, MEM-LS, AMC-R)	NA	NA	NA	Relapse
4* (hospital H)	Dysuria, renal calculi; patient had received unknown duration of AMC from a referral hospital	−3 (urine)	DR80110A (AMC-R)	CAZ	0	13	Discharged at day 13 with home oral SXT
				SXT	10	13	
5* (hospital I)	Fever, history of foot wound exposure to soil and carotid space abscess; patient was diagnosed with hypertensive emergency, cardiomegaly, and pulmonary congestion	1 (blood)	DR90031A (S)	CTR	0	1	Discharged at day 6 with home oral SXT and AMC
				AMC	1	6	
	Relapsed infection	520 days after first episode (pus)	DR90031E (MEM-LS)	NA	NA	NA	Relapse
6 (hospital I)	History of melioidosis 2 yrs earlier, fever, and splenic abscess	1 (blood)	DR90049A (MEM-LS)	CTR	0	2	Discharged at day 15 with home oral SXT
				DOX	0	2	
				CAZ	2	15	

a MDR, multidrug resistant; TB, tuberculosis; MEM, meropenem; AMC, amoxicillin-clavulanic acid; CFZ, cefazolin; CAZ, ceftazidime; AZM, azithromycin; SXT, trimethoprim-sulfamethoxazole; CST, colistin; VAN, vancomycin; STR, streptomycin; CTR, ceftriaxone; DOX, doxycycline; S, susceptible; LS, less susceptible; R, resistant; NA, not available; *, transferred from referring hospitals.

### Genome analysis of genes associated with antibiotic resistance.

Whole genomes of primary CAZ-R, MEM-LS, and AMC-R isolates DR10212A, DR30013A, DR80110A, and DR90049A and relapse pair isolates DR50054A-DR50054E and DR90031A-DR90031E were sequenced to define multilocus sequence types (MLST) and searched for known and new mutations in β-lactam resistance-associated genes, including β-lactamase genes (*penA*, *oxa*) and genes coding for a β-lactam target (PBP3), efflux pump systems, and outer membrane porin (Table 5 and see Table S2 in the supplemental material). We obtained > 90% sequence coverage of the K96243 reference genome for WGS of all isolates.

**TABLE 5:**
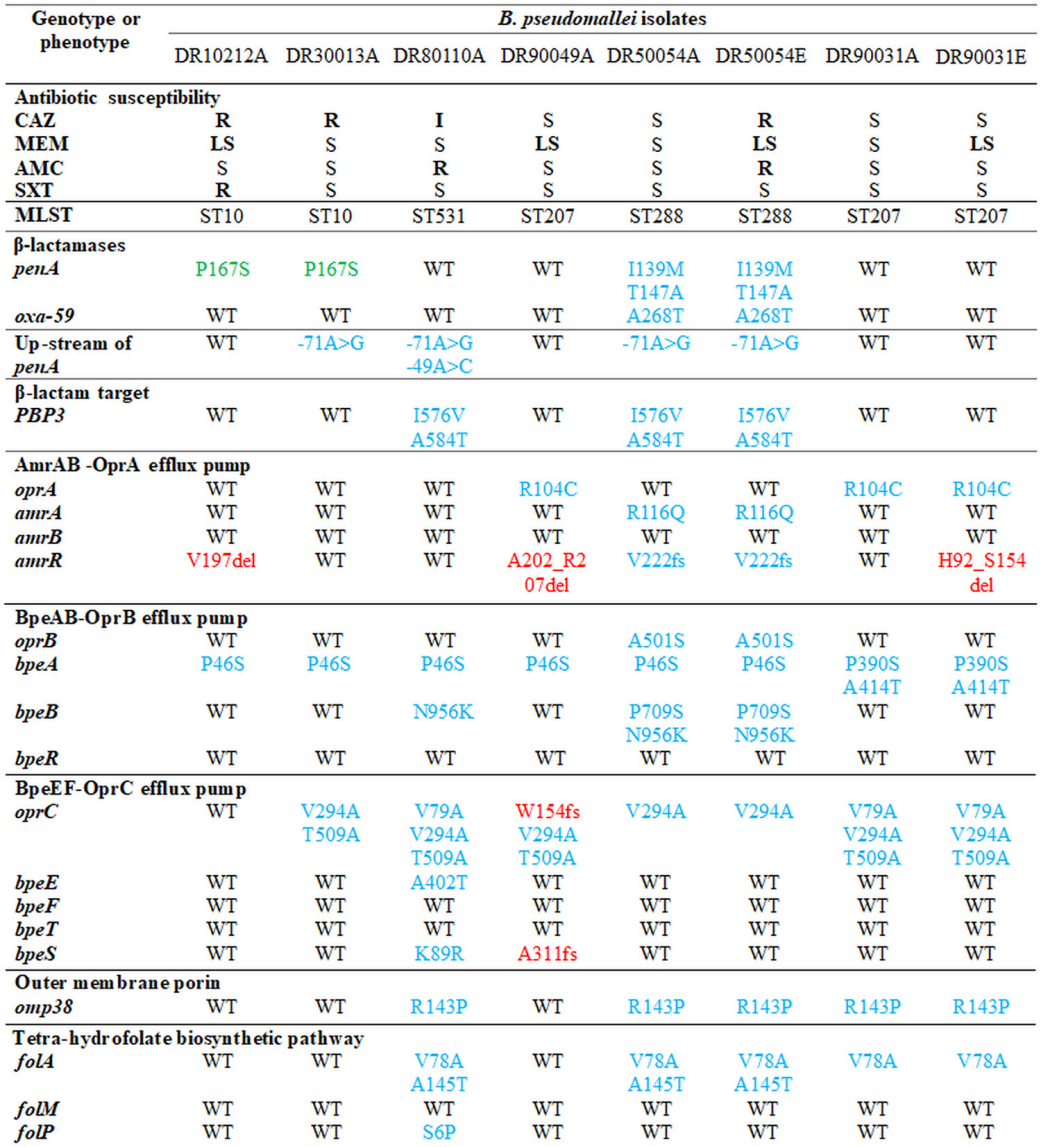
Whole-genome analysis of antibiotic resistance-associated genes in B. pseudomallei isolates^*a*^

a Mutations were amino acid changes due to single nucleotide polymorphism, deletion, and frameshift mutation observed in B. pseudomallei isolates with decreased antibiotic susceptibility compared to K96243A. A, primary isolate; E, relapse isolate; MLST, multilocus sequence type; WT, wild type; fs, frameshift mutation; del, deletion; S, susceptible. Boldface in antibiotic susceptibility indicates resistance (R), less susceptible (LS), or intermediate (I). The mutations colored in blue were also found in antibiotic-susceptible B. pseudomallei isolates. Those colored in green were known CAZ-R variants (20, 22). Novel mutations found in this study are colored in red.

The MLST of both DR10212A and DR30013A was ST10, which was identical to the ST of K96243. The MLST of DR80110A was ST531. DR50054A had the same ST288 as the DR50054E relapse pair isolate. DR90049A shared the identical ST207 with relapse isolate pair DR90031A and DR90031E.

WGS analysis of β-lactam resistance-associated genes revealed a *penA* mutation in three isolates with CAZ-R phenotype (DR10212A, DR30013A, DR50054E) and a CAZ-S isolate (DR50054A) (Table 5). DR10212A and DR30013A with P167S at the PenA omega loop were also documented in CAZ-R isolates in previous works (20, 22, 27). DR50054A and DR50054E shared the same PenA mutations of I139M and T147A. DR80110A with CAZ-I, DR90049A, and paired isolates DR90031A and DR90031E with MEM-LS bore wild-type (WT) PenA. We observed a single-nucleotide polymorphism (SNP) of −71A>G at the upstream promoter region of *penA* in DR30013A, DR80110A, DR50054A, and DR50054E. In DR80110A, there was an additional −49A>C located upstream of *penA*. Analysis of *oxa* demonstrated that all of the isolates had Oxa-59 identical to K96243 (WT) except for DR50054A and DR50054E, where the amino acid substitution A268T occurred in both (Table 5).

**FIG 2:**
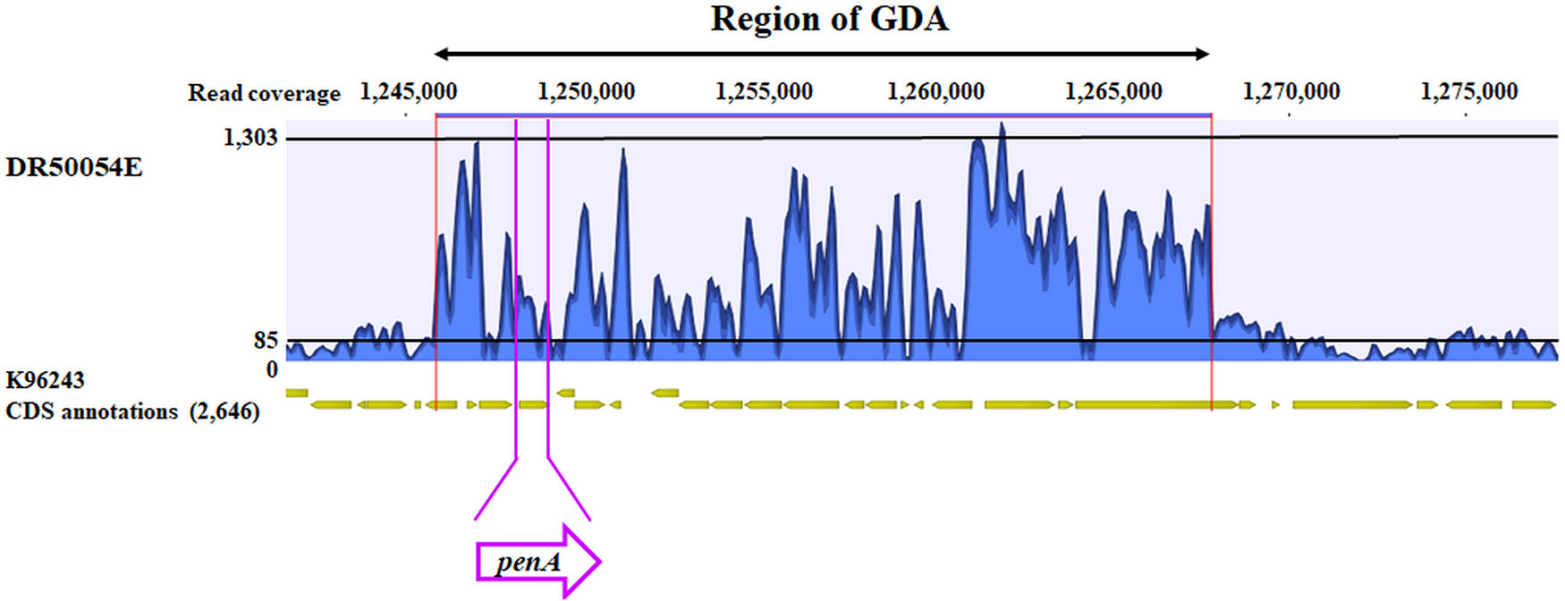
Gene duplication and amplification (GDA) in clinical Burkholderia pseudomallei strain DR50054E. The 22-kb amplified regions in chromosome 2 of relapse strain DR50054E involve 20 genes from *BPSS0944*, *BPS_RS23855* to *BPSS0960*, and *BPS_RS23950* (red line) (see Table S3 in the supplemental material), including *penA* (purple line). The average sequence read coverage of this GDA region is approximately 6-fold higher than the average genome coverage.

No large deletion was observed in the β-lactam target PBP3 (*BPSS1219*), but we found two SNPs affecting PBP3 (I576V and A584T) in DR80110A and isolate pair DR50054A-DR50054E (Table 5). Interestingly, we observed in the MDR isolate DR50054E a notable increase of read coverage of approximately 6-fold involving 22-kb regions (*BPSS0944*; NCBI new locus tags *BPS_RS23855* to *BPSS0960*; NCBI new locus tag *BPS_RS23950*) containing *penA* and 19 other genes in chromosome 2 compared to the average genome coverage (Fig. 2) (Table S3). This feature was not detected in the first-episode isolate DR50054A and other resistant isolates.

We next examined the genetic alteration related to RND multidrug efflux pumps AmrAB-OprA, BpeAB-OprB, and BpeEF-OprC. We observed that DR10212A had a deletion at *amrR* (V197del) of the AmrAB-OprA efflux pump (Table 5). In strain DR90049A, we observed a nonsynonymous SNP (R104C) in OprA and an in-frame deletion of 6 amino acids (A202_R207del) in AmrR. Isolates DR50054A and DR50054E had an 11-bp deletion in *amrR* which conferred a frameshift at V222 (V222fs) and an SNP in *amrA* resulting in amino acid change R116Q. Compared to primary isolate DR90031A, the relapse isolate DR90031E with an MEM-LS phenotype had an in-frame deletion of 63 amino acids from H92 to S154, shortening AmrR to 160 residues. Both DR90031A and DR90031E also contained R104C in OprA, which was also observed in DR90049A.

For the BpeAB-OprB efflux pump, except for isolates DR90031A and DR90031E, P46S was found in BpeA of the other six isolates: DR10212A and DR30013A (CAZ-R), DR90049A (MEM-LS), and DR80110A (AMC-R) isolates and relapse pair isolates DR50054A and DR50054E (Table 5). P46S was the only variant identified in DR10212A. DR80110A shared BpeB N956K with DR50054A and DR50054E. Four SNPs resulting in amino acid substitutions of A501S in OprB, P46S in BpeA, and P709S and N956K in BpeB were common SNPs between the first isolate DR50054A and relapse pair isolate DR50054E. Two SNPs in *bpeA* resulting in amino acid changes P390S and A414T were found in both DR90031A and DR90031E.

For the BpeEF-OprC efflux pump, DR10212A carried *oprC*, *bpeE*, *bpeF*, *bpeT*, and *bpeS* WT genes. We observed amino acid alterations of V294A (7 isolates), T509A (5 isolates), V79A (3 isolates), and W154fs (1 isolate) in OprC, a A402T variant in BpeE (1 isolate), and in BpeS, K89R (1 isolate) and A311fs (1 isolate) (Table 5).

A gene encoding the outer membrane porin Omp38 was also examined. An SNP in *omp38* (R143P) was detected in DR80110A, DR50054A, DR50054E, DR90031A, and DR90031E (Table 5).

### Identification of genetic mechanisms for antibiotic resistance.

To exclude unrelated resistance mechanisms, we next investigated whether SNPs in β-lactam resistance-associated genes and the Omp38 porin gene (Table 5) were present in genomes of CAZ-S, MEM-S, and AMC-S isolates (*n* = 697; BioProject accession no. PRJEB25606) in our data set. Most mutations were detected in antibiotic-susceptible isolates. However, we observed some alterations involving only CAZ-R and MEM-LS strains, including P167S in PenA, H92_S154del, V197del, and A202_R207del in AmrR, W154fs in OprC, and A311fs in BpeS. Further examination of genes affecting SXT susceptibility of SXT-R isolate DR10212A revealed no mutation in *folA*, *folM*, and *folP*, which are involved in the tetrahydrofolate biosynthetic pathway (Table 5).

We hypothesized that alterations in PenA might have affected CAZ, MEM, and AMC susceptibility in B. pseudomallei isolates presenting with an MDR phenotype. Hence, in this study, we focused on the evaluation of the *penA* role in MDR strains DR10212A and DR50054E with decreased susceptibility toward β-lactam antibiotics. The SNPs found affecting *penA* were verified by Sanger sequencing.

### Mutagenesis of *penA* in MDR strain DR10212A.

**FIG 3:**
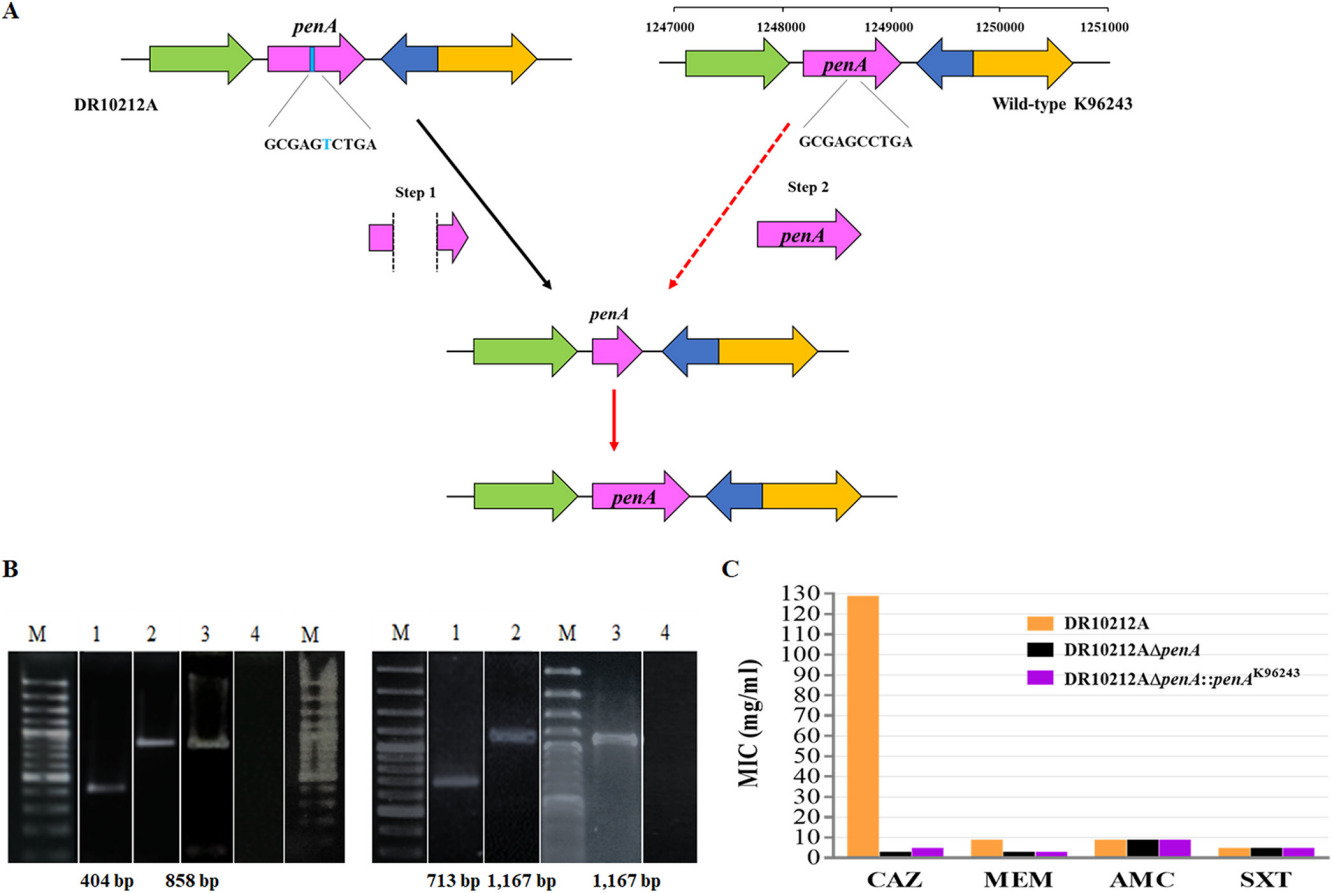
Construction of *penA* mutants in Burkholderia pseudomallei strain DR10212A. (A) Construction of DR10212AΔ*penA* and DR10212Δ*penA*::*penA*^K96243^. *penA* is also known as *BPSS0946* (*BPS_RS23870*) (pink). The gene located upstream of *penA* is *BPSS0945* (*BPS_RS23865*) (green). The downstream genes are NCBI recently annotated hypothetical gene *BPS_RS23875* (dark blue) and *BPSS0948* (*BPS_RS238800*) (yellow). In step 1, *penA* encoding P167S (light blue) was knocked out from parental strain DR10212A and replaced with wild-type *penA* from K96243 in step 2 to produce DR10212AΔ*penA*::*penA*^K96243^. Briefly, fragments containing the desired *penA* deletion and insertion sequences were synthesized. The plasmid vectors containing the cloned regions of homology allowed the exchange and integration of desired fragments into the chromosome by homologous recombination between cloned and chromosomal sequence. Multiple counterselection markers were applied using kanamycin and sucrose to finally obtain a desired clone. (B) The correct size of the products was confirmed by PCR using primer penA_muta (left) and penA1 (right). Lanes: M, 100-bp plus ladder; 1, DR10212AΔ*penA*; 2, DR10212AΔ*penA*::*penA*^K96243^; 3, K96243; 4, sterile water. The gels were sliced for labeling purposes. (C) The MICs of CAZ for DR10212A (orange), DR10212AΔ*penA* (black), and DR10212AΔ*penA*::*penA*^K96243^ (purple) were 128 μg/ml (R), 2 μg/ml (S), and 4 μg/ml (S), respectively. After complementation with wild-type *penA* from K96243, the MIC of MEM dropped from 8 μg/ml (DR10212A, LS) to 2 μg/ml (DR10212AΔ*penA* and DR10212AΔ*penA*::*penA*^K96243^, S). The MICs of all isolates toward AMC and SXT remained unchanged, showing AMC-S and SXT-R phenotypes.

Both DR10212A and DR30013A had PenA P167S. DR30013A was CAZ-R, while DR10212A was CAZ-R, MEM-LS, and SXT-R (Table 3). PenA P167S has been reported previously to confer resistance to CAZ but not MEM (20, 27) (Table S2), as observed in DR10212A. To evaluate the potential role of PenA in MEM susceptibility in addition to the well-known role of PenA in CAZ susceptibility, we proceeded with an investigation of MDR strain DR10212A. First, we successfully created a *penA* deletion mutation in the parental strain, DR10212A, to observe the loss of the resistance phenotype. DR10212AΔ*penA*::*penA*^K96243^ was generated by complementation of wild-type K96243 *penA* into DR10212AΔ*penA* (Fig. 3A).

PCR screening using two primer pairs to cover the whole *penA* sequence (Table S1) confirmed the generation of *penA* deletion and wild-type K96243 *penA* insertion. DR10212AΔ*penA* showed smaller *penA* fragments, 404 bp and 713 bp, upon deletion of the region containing the SNP than those of DR10212AΔ*penA*::*penA*^K96243^ and wild-type K96243 *penA*, which were 858 bp and 1,167 bp (Fig. 3B). DNA sequencing analysis confirmed the correct DR10212AΔ*penA* and DR10212AΔ*penA*::*penA*^K96243^ sequences.

### Antibiotic susceptibility of DR10212A mutant and complemented strains.

We observed that DR10212AΔ*penA* reduced the MIC of CAZ by 64-fold from 128 μg/ml (resistant) to 2 μg/ml (susceptible) and the MIC of MEM by 4-fold from 8 μg/ml (less susceptible) to 2 μg/ml (susceptible) while maintaining the same MICs for AMC (8/4 μg/ml, susceptible) and SXT (4/76 μg/ml, resistant) (Fig. 3C). DR10212AΔ*penA*::*penA*^K96243^ was CAZ-S, showing an MIC of 4 μg/ml, similar to that of the *penA*-contributing strain K96243, and MEM-S, with an MIC of 2 μg/ml. Due to concerns about manipulating a possible bioterrorism agent, construction of a resistant phenotype (P167S) by introduction of 517C>T from a resistant strain into the original susceptible parental strain, K96243, was not done. Our data suggest that P167S in PenA is the mechanism responsible for CAZ-R and MEM-LS, but not SXT-R, for DR10212A.

### Growth curve analysis.

**FIG 4:**
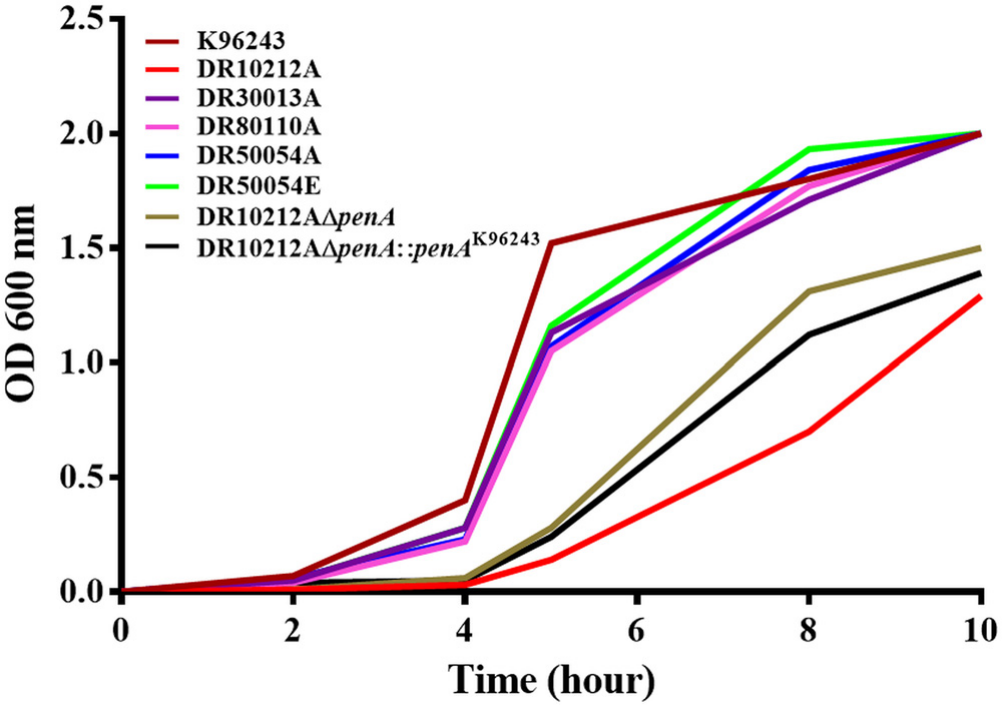
Growth curves of Burkholderia pseudomallei isolates cultured in LB broth.

Growth rate could be a determining factor for decreased β-lactam susceptibility, as β-lactams act on metabolically active cells. We observed a clear gap in the growth of DR10212A and its derivative mutants in LB broth; these strains exhibited a longer lag phase and slower growth than the other strains (Fig. 4). Three strains (DR10212A, DR10212AΔ*penA*, and DR10212AΔ*penA*:: *penA*^K96243^) required an additional 2 h to reach mid-log phase before proceeding to *penA* transcriptional analysis and PenA β-lactamase activity, as described in the next sections.

### Quantification of *penA* transcript levels.

**FIG 5:**
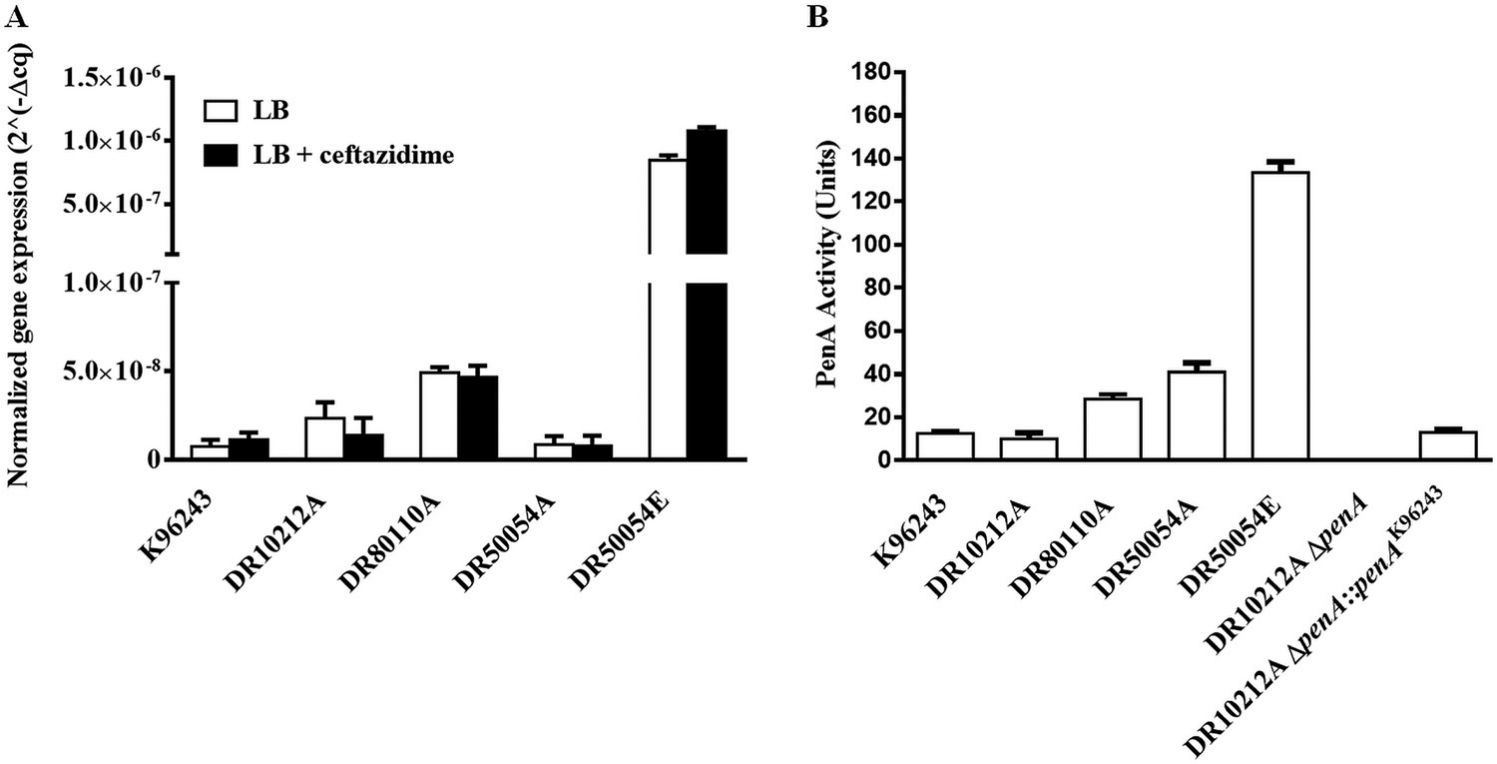
Expression of *penA* and β-lactamase activity of clinical Burkholderia pseudomallei isolates and laboratory-generated *penA* mutants. (A) RT-PCR analysis was performed to compare the levels of *penA* expressed. The strains used were K96243 (CAZ-S), DR10212A (CAZ-R), DR80110A (CAZ-I), and primary-relapse pair DR50054A (CAZ-S) and DR50054E (CAZ-R). Briefly, bacteria cells were grown to mid-log phase in LB broth. Equal portions of the cultures remained untreated or were treated with a subinhibitory concentration of ceftazidime at 32 μg/ml. Total RNA extraction was conducted on bacterial cells at mid-log phase. The *penA* gene expression levels shown were after normalization with 16S rRNA. The error bars indicate the standard deviations between results of two biological replicates. (B) PenA activity for isolates DR10212A, DR80110A, DR50054A, DR50054E, and DR10212AΔ*penA*::*penA*^K96243^ grown in LB broth. The enzyme activity was analyzed using nitrocefin as the reporter substrate. The data were analyzed based on two individual experiments conducted on separate days. The positive and negative controls were K96243 and DR10212AΔ*penA*, respectively. PenA β-lactamase activity units were derived by subtracting the activity observed with DR10212AΔ*penA*.

To investigate whether the GDA event found in DR50054E is associated with elevated gene expression, a RT-PCR assay based on *penA* was employed under two culture conditions: LB broth and LB broth containing CAZ. The PenA wild-type and variant isolates were assessed based on their *penA* expression profile. Indeed, DR50054E expressed a high level of *penA* transcripts (Fig. 5A), suggesting that the overexpression was associated with GDA (Fig. 2). Surprisingly, a CAZ-I and AMC-R isolate, DR80110A, displayed relatively higher levels of *penA* transcripts than the CAZ-R isolate DR10212A. K96243, which served as the reference strain, showed a *penA* expression level similar to that of DR50054A. For CAZ induction analysis in CAZ-susceptible and -resistant strains, we compared the expression levels of *penA* before and after CAZ treatment. There was no significant differential *penA* expression level observed between these two conditions (Fig. 5A).

### PenA β-lactamase activity.

To validate the genetic mechanisms of resistance to β-lactams and *penA* expression levels, we determined PenA β-lactamase activity for DR10212A, DR80110A, DR50054A, DR50054E, and DR10212A mutant derivative DR10212AΔ*penA*::*penA*^K96243^. Strains K96243 and DR10212AΔ*penA* were used as positive and negative controls, respectively. Consistent with the high *penA* expression level observed in DR50054E grown in LB broth, high PenA β-lactamase activity was also detected in DR50054E (Fig. 5B). This observation further confirmed the elevated resistance due to GDA in DR50054E. Confirming the *penA* transcript result, DR80110A had higher β-lactamase activity than that of K96243 and DR10212A.

## DISCUSSION

CAZ is commonly used for melioidosis treatment, and MEM is reserved for severe cases in Thailand and many countries. Our data indicate that the prevalence of primary resistance to β-lactams is low, with CAZ-R and MEM-LS at 0.15% and AMC-R at 0.08%. Two of 13 relapse isolates exhibited decreased β-lactam susceptibility: CAZ-R, 7.7%; MEM-LS, 15.4%; and AMC-R, 7.7%. All patients who exhibited CAZ-R (*n* = 3) and AMC-R (*n* = 2) had received CAZ and AMC prior to or during their treatment course, respectively. Here, we described two clinical MDR strains of B. pseudomallei and two fatal cases involving PenA alterations. WGS analysis identified an amino acid substitution of P167S in PenA and GDA events involving *penA*, the most prominent mechanisms of high CAZ MIC. In addition, we observed new mutations upstream of *penA* −49A>C relating to increased *penA* expression and in genes encoding AmrAB-OprA and BpeEF-OprC efflux pump systems that might be involved in decreased susceptibility to MEM.

DD is commonly used for antibiotic susceptibility testing (AST) in resource-limited hospitals instead of the laborious standard BMD or the costly Etest and due to the limited availability of automated systems (33, 34). Despite the unavailability of interpretive guidelines for DD at the CLSI, zone diameters for related organisms such as Pseudomonas aeruginosa and *Enterobacterales* (35) are applied, with the exception of those for SXT (12–14, 16, 33). Interpretation of SXT susceptibility by disk diffusion could be misleading due to the difficulty to read the diffuse edges, and hence, BMD or the Etest is recommended (24, 34). Due to its ease of use, the Etest has been applied in laboratories instead of BMD (13, 15, 33, 36–38). By the DD method, MEM and AMC resistance might be overreported, as previously described (see Table S4 in the supplemental material). Our data in this study demonstrated misinterpretation by DD in hospital A and hospital H for AMC-I and MEM-R, respectively (Table 2), suggesting that DD should be repeated and MIC-based BMD should be conducted as a confirmatory test for resistant or intermediate results. Hospital I reported several IPM-R and MEM-R isolates. Investigations into the hospital reports pointed out that Vitek 2 AST cards misidentified B. pseudomallei as Burkholderia cepacia, *Pseudomonas* sp., or Pseudomonas aeruginosa and thus required an extra step to reconfirm B. pseudomallei identification by Vitek 2 GN ID card. It was noted that the MIC values generated by Vitek 2 AST cards for these isolates were within the susceptible range of the CLSI (39), but the automated interpretation of the Vitek 2 system (advanced expert system) misinterpreted them as resistant. Therefore, readers may refer to the CLSI guidelines to interpret the antibiotic susceptibility based on the MIC values when Vitek 2 AST cards are used for AST. Misidentification of resistant phenotypes could lead to a fatal outcome when an effective drug is not used. Strict monitoring in a timely manner of B. pseudomallei antibiotic susceptibility is required to avoid inappropriate administration of antibiotics.

The low number of isolates with primary CAZ and MEM resistance in this study was comparable to earlier reports from Thailand (CAZ-R, 0.1 to 1.5%; MEM-R, 2%) (12–14). Primary CAZ resistance in Malaysia was reported at 0.6 to 2.4% (15–17), while Australia (36), Cambodia (37, 38), and Vietnam (40) reported no primary resistance to CAZ or MEM (Table S4). In this study, we found two primary MEM-LS isolates in Thailand, which is worrisome. Previous studies based on the Etest reported that all MEM-susceptible isolates had an MIC of ≤2 μg/ml (13, 15, 36, 37), but one report showed 2% primary MEM-R by DD in Thailand (12). However, analysis by DD may be an overrepresentation, and we used standard BMD as a confirmation test in this study. We observed delayed MEM therapy in one fatal case (patient 2) involving a CAZ-R isolate, which indicates the importance of initial AST screening for appropriate antibiotic therapy. Unfortunately, another fatal melioidosis case (patient 1) was coinfected with MDR Acinetobacter baumannii. The patient probably acquired CAZ-R and MEM-LS during therapy and showed no improvement upon AMC treatment despite isolate DR10212A being AMC-S. In addition, DR10212A had a lower growth rate, which could be a trade-off for fitness advantage to survive against β-lactams, which kill metabolically active cells. A further study is required to investigate the virulence of this strain.

All isolates were susceptible to IPM, suggesting a favorable alternative, and cross-resistance between meropenem and imipenem may not occur (30). Yet, we noted that the absence of IPM-R could be due to infrequent IPM use and therefore no antibiotic selection pressure. We also recorded that for two relapse cases with decreased susceptibility to CAZ, MEM, or AMC, to which they were originally susceptible, both patients received either i.v. AMC plus oral AMC and SXT or i.v. CAZ plus oral AMC treatments during the first episode. The use of second-line AMC (7) as treatment may be ineffective for bacterial clearance. Moreover, MEM-LS isolates were recovered from both patients 3 and 5 despite their never being treated with MEM in the initial treatment course, also postulating the possible occurrence of cross-resistance to AMC, ceftriaxone, or TB drugs such as azithromycin and streptomycin received during the therapy.

Our study indicated that both previously described PenA P167S (20, 22) and GDA (21, 26) events are common CAZ-R-conferring mechanisms of B. pseudomallei isolated from patients during and after treatment with CAZ (Table S2). In contrast to findings for PenA T147A, which has been shown to confer resistance to both AMC and IPM (25), we shared a similar observation as Sarovich et al. that T147A by itself is not associated with AMC and IPM resistance (30). A search in our whole-genome database indicated that both PenA I139M and T147A observed in relapse pairs DR50054A and DR50054E are commonly found in B. pseudomallei isolates susceptible to CAZ, MEM, and AMC, and therefore these mutations may not be significantly associated with resistance in DR50054E.

No other mutation besides two SNPs at position 49 (−49A>C) and 71 (−71A>G) upstream of *penA* were observed in CAZ-I isolate DR80110A. Since −71A>G is commonly observed in CAZ-S B. pseudomallei isolates in our data set, the SNP 49A>C might be the factor leading to increased *penA* expression in strain DR80110A. This mutation could have interfered with the terminator function of the *penA* transcriptional terminator (rho-independent TERM264) situated between nucleotides −25 and −73 upstream of *penA* (41). Although infrequent, this mutation was also detected in 1.4% of CAZ-S isolates in our data set, suggesting that this SNP, −49A>C, probably reduced bacterial susceptibility to CAZ but was insufficient to result in CAZ resistance.

PBP3 deletion is associated with the CAZ-R phenotype and growth defect (19). Although DR50054A, DR50054E, and DR80110A had I576V and A584T amino acid changes in PBP3, these mutations were also observed in CAZ-S isolates and hence are unlikely to be associated with CAZ-R. We noted that DR50054A, DR50054E, and DR80110A were able to grow normally in LB broth, comparable to K96243 (Fig. 4).

The TetR-type regulator gene *amrR* acts as the repressor of the AmrAB-OprA efflux pump and is responsible for efflux pump overexpression and elevated MEM MIC (30, 31). The new *amrR* mutation includes deletions at two different positions leading to H92_S154del and A202_R207del in strains DR90049A and DR90031E and are possibly associated with the MEM-LS phenotype. LysR-type regulators genes *bpeS* and *bpeT* act as transcriptional activators of the BpeEF-OprC efflux pump together with the tetrahydrofolate biosynthetic pathway: *folA*, *folM*, and *folP* contribute to the SXT-R phenotype (21, 32). However, we observed no alteration of these genes in SXT-R isolate DR10212A. Instead, new frameshift mutations in *oprC* (W154fs) and *bpeS* (A311fs) were observed in MEM-LS DR90049A, suggesting a possible connection between the MEM-LS phenotype and the BpeEF-OprC efflux pump (24, 30). The mechanism related to the SXT-R profile in DR10212A could be due to AmrAB-OprA V197del or mechanisms other than the BpeEF-OprC efflux pump and the tetrahydrofolate biosynthetic pathway (30, 32).

In addition to CAZ-R, we associated the decreased MEM susceptibility in DR10212A with PenA P167S, as our data showed that upon deletion or replacement of *penA* in DR10212A (128 μg/ml) with wild-type *penA*^K96243^, the MICs of both CAZ and MEM dropped into the susceptible category. The CAZ MIC decreased by 64-fold and 32-fold in DR10212AΔ*penA* (2 μg/ml) and DR10212AΔ*penA*::*penA*^K96243^ (4 μg/ml), respectively. The MEM MIC decreased by 4-fold to 2 μg/ml in both mutants. Both AMC and SXT MICs were unaltered following *penA* replacement. Taken together, the data suggest that PenA P167S was responsible for the CAZ-R and MEM-LS phenotypes in DR10212A. Our observation was in concordance with previous reports that PenA P167S confers the CAZ-R phenotype (20, 22, 27, 42) but differed in the second notion that P167S also resulted in reduced susceptibility to MEM. To start with, the parental MDR strain DR10212A had higher MICs of CAZ and MEM, at 128 μg/ml and 8 μg/ml, than the 64 μg/ml and 1 μg/ml of DR30013A and a clinical isolate from P45 (Australia), both containing PenA P167S (20). The apparently higher MIC value might be due to the relatively slower growth of DR10212A than of DR30013A. Another Thai clinical isolate, 316c, bearing the PenA P167S variant also had a CAZ MIC of 64 μg/ml (22). Despite the lower MIC of CAZ at ≥32 μg/ml, Ho et al. demonstrated that strain BPLH-1-2 with PenA P167S generated upon selection on increasing CAZ concentrations had a 2-fold-elevated MEM MIC, to 4 μg/ml (MEM-LS), compared to that of the wild-type strain, BPLH-1 (43). However, a laboratory-generated PenA P167S mutant, Bp82.5, exhibited an increased MIC of CAZ from 3 μg/ml to 16 to 24 μg/ml and an unchanged MIC of MEM at 0.5 to 0.75 μg/ml in comparison to that of the parental strain, Bp82 (27). Our experiment differed from that of Rholl et al. in that we replaced the PenA P167S with wild-type PenA K96243 in parental strain DR10212A instead of introducing the mutation into laboratory strain Bp82 (27).

Occurrence of a GDA event was previously reported in strain MSHR5654 and the isogenic pair of strains MSHR8441 and MSHR8442 from patients in Australia (21) and in Bp5041c from a patient in Thailand (26). A GDA event was observed in Bp5041c isolated after 15 days of CAZ therapy (26). Yet, in our study, DR50054E, which presented with GDA, was isolated 341 days after completed CAZ therapy. Similarly, MSHR5654 with GDA was isolated approximately 25 months after completion of CAZ and MEM therapy (21). In addition to a GDA event involving the *penA* region, MSHR5654 also had PenA C69Y (Table S2) associated with high CAZ-R (18, 21, 23) and a BpeT T314fs conferring SXT-R (21, 31). In DR50054A and DR50054E (CAZ-R, MEM-LS, AMC-R), there were no gene mutations observed for *oxa-59*, PBP3, or efflux pumps differentiating this pair except for the GDA event.

Our study confirmed that the presence of CAZ in culture medium for 2 h had no effect on the CAZ susceptibility and *penA* expression level in comparison with isolates cultured in CAZ-untreated medium, consistent with the report of Rholl et al. (27).

Alterations involving GDA in CAZ-R isolate DR50054E and −49A>C upstream of *penA* in CAZ-I isolate DR80110A resulted in increased *penA* expression level and β-lactamase activity. However, the *penA* expression level and β-lactamase activity do not correlate well with the CAZ MIC level observed in DR10212A and DR50054A. Lower *penA* expression level and β-lactamase activity were observed in DR10212A despite showing a high CAZ MIC. It is likely that in DR10212A, the P167S substitution at the omega loop of PenA led to increased affinity for CAZ compared to wild-type PenA, resulting in high CAZ resistance (22, 43). It was also observed in CTX-M-14 and CTX-M-19 of extended-spectrum β-lactamase (ESBL) *Enterobacteriaceae* harboring P167S that the mutation affects the binding and interaction with the aminothiazole ring of CAZ, resulting in enhanced CAZ hydrolysis (42, 44).

PenA alterations could be related to decreased CAZ and probably MEM susceptibility. Mutation detection focusing on PenA SNPs including the promoter region would be valuable in clinical settings, prompting a change to alternative drugs such as IPM to alleviate the possibility of treatment failure due to ineffective antibiotics. For instance, rapid identification of PenA P167S using real-time PCR and an RNA-based triplex qPCR assay targeting upregulation of AmrAB-OprA, BpeEF-OprC, and BpeAB-OprB efflux pumps have been developed by Sarovich et al. and might be useful (20, 31).

The determinant for AMC resistance is still unclear. Further assessment of mechanisms besides enzymatic inactivation by PenA in this study would be beneficial. Currently, we are in the process of evaluating the putative mutations involving efflux pumps mechanism, namely, in *amrR* (H92_S154del, V197del, A202_R207del), *oprC* (W154fs), and *bpeS* (A311fs), which possibly give rise to MEM-LS and SXT-R.

### Conclusions.

The low prevalence of B. pseudomallei isolates with resistance to β-lactams *in vitro* suggests the appropriateness of CAZ and carbapenem treatment regimens. Alterations affecting *penA*, including SNPs, gene duplication events, and the upstream promoter region, are currently the major determinants corresponding to decreased CAZ susceptibilities. In addition to the efflux pump system, PenA may extend its role to affect MEM susceptibilities. Further validation of the efflux pump mechanism observed in MEM-LS and SXT-R isolates is required to ascertain their phenotypes. The mortality rate of melioidosis still remains high, raising questions about the association of other determinants with regard to patient outcomes; however, the unfilled gap leading to treatment failure requires further investigation.

## MATERIALS AND METHODS

### Ethical approval.

The Ethics Committee of the Faculty of Tropical Medicine, Mahidol University (approval number MUTM2015-002-05), has approved this study and the consent procedure. Written informed consent was obtained from all participants or their representatives.

### Biosafety approval.

This study was approved by the Institutional Biosafety Committee, Faculty of Tropical Medicine, Mahidol University (approval number FTM-IBC-21-01).

### Bacterial isolates.

A total of 1,317 clinical B. pseudomallei isolates were obtained from 1,304 patients in nine hospitals in northeast Thailand between July 2015 and December 2018. These included 1,304 primary isolates and 13 isolates from relapse cases. Primary and relapse isolates were denoted by “A” and “E,” respectively, at the end of the strain name. The hospitals (numbers of isolates) were as follows: hospital A (*n* = 227), hospital B (*n* = 89), hospital C (*n* = 26), hospital D (*n* = 140), hospital E (*n* = 226), hospital F (*n* = 198), hospital G (*n* = 127), hospital H (*n* = 177), and hospital I (*n* = 107). The isolates were identified as B. pseudomallei at the hospitals and confirmed at the Faculty of Tropical Medicine, Mahidol University (FTM), by a specific exopolysaccharide-monoclonal-based latex agglutination assay (45) and matrix-assisted laser desorption ionization–time-of-flight mass spectrometry (MALDI-TOF MS) (46). The source and site of the specimens varied, from blood (*n* = 940), respiratory secretions (*n* = 133), body fluid (*n* = 44), pus (*n* = 164), urine (*n* = 26), wound swab (*n* = 3), and tissue (*n* = 7) (Table 1). The bacteria were stored in tryptic soy broth (TSB) with 20% glycerol at −80°C until use. All B. pseudomallei cultures were performed in a biosafety level 3 (BSL3) laboratory.

### Determination of antibiotic susceptibility.

Four different procedures were used by the nine hospitals for antibiotic susceptibility testing (AST) (Fig. 1). Hospitals A, B, E, and H used DD only; hospitals C, F, and G used a combination of DD and Etest; hospital D used disk DD, BMD, and the BD Phoenix automated microbiology system (BD Diagnostics Systems); and hospital I used Vitek 2 system (bioMérieux). At FTM, we performed AST for clinical B. pseudomallei isolates either for which AST had not been performed or which had been reported as intermediate or resistant by the hospitals. B. pseudomallei isolates were cultured on Mueller-Hinton agar (MHA) (Oxoid) or cation-adjusted Mueller-Hinton broth (CAMHB) (Sigma). Antibiotic susceptibility was tested by DD and confirmed with BMD. The antibiotic discs used were CAZ, IPM, MEM, and AMC (Oxoid). The antibiotic concentration ranges used for BMD were as follows: CAZ, 0.25 to 256 μg/ml; MEM, 0.03 to 32 μg/ml; AMC, 0.06/0.03 to 64/32 μg/ml; and SXT, 0.03/0.57 to 32/608 μg/ml (Sigma). Standard MIC panels were prepared with CAMHB containing the serial drug dilutions with a final volume of 100 μl per well.

Bacterial suspensions in 0.85% sodium chloride were prepared from 18 h of culture on Columbia agar (CA) to achieve a target concentration of approximately 1 × 10^8^ CFU/ml for DD and 5 × 10^5^ CFU/ml for BMD. The results were read after 18 h of incubation at 37°C. CLSI guidelines were used for the interpretation of both DD and BMD. The threshold zone sizes of *Enterobacterales* and Pseudomonas aeruginosa were applied as a reference for DD (35). For BMD, Escherichia coli ATCC 25922, E. coli ATCC 35218, P. aeruginosa ATCC 27853, and B. pseudomallei K96243 were used as controls. MIC reference categories for B. pseudomallei are available for CAZ, AMC, and SXT (39) but not for MEM; hence, we considered the epidemiological cutoff value (ECOFF) of >2 μg/ml as the breakpoint to differentiate strains with decreased MEM susceptibility from the wild type (47).

### Whole-genome sequencing.

The whole genomes of B. pseudomallei isolates were sequenced using an Ion Torrent or Illumina platform (Illumina MiSeq or HiSeq 2000) at the Center for Medical Genomics, Faculty of Medicine, Ramathibodi Hospital, Bangkok, Thailand, and the Wellcome Sanger Institute, United Kingdom. Briefly, the genomic DNA was extracted from 1.5 ml of overnight bacterial culture in LB broth using a QIAmp DNA minikit (Qiagen, Germany). The DNA libraries were prepared for a 150-bp read with an Ion Xpress Plus fragment library kit (Life Technologies) for the Ion Torrent system and for 75- or 250-bp paired-end-reads libraries for the Illumina system. The short reads produced from both platforms were mapped to the reference B. pseudomallei K96243 genome (NC_006350.1, NC_006351.1) using CLC Genomic Workbench version 20.0 (CLC Bio-Qiagen). Multilocus sequence type was analyzed using https://pubmlst.org/.

### Antibiotic resistance-conferring genes.

Whole-genome searches were performed on the antibiotic resistance-associated genes described previously (5, 48), including β-lactamase genes *penA* (*BPSS0946*; *BPS_RS23870*) and *oxa* (*BPSS1997*; *BPS_RS29690*), β-lactam drug target *PBP3* (*BPSS1219*; *BPS_RS25365*), RND multidrug efflux systems and respective regulators AmrAB-OprA (*BPSL1802-BPSL1804*; *BPS_*RS09570-RS09580), *amrR* (*BPSL1805*, *BPS_RS09585*), BpeAB-OprB (*BPSL0814-BPSL0816*; *BPS_*RS04290-RS04300), *bpeR* (*BPSL0812*, *BPS_RS04280*), BpeEF-OprC (*BPSS0292-BPSS0294*; *BPS_*RS20225-RS20235), *bpeT* (*BPSS0290*, *BPS_RS20215*), and *bpeS* (*BPSL0731*, *BPS_RS03845*), tetrahydrofolate biosynthetic pathway genes *folA* (*BPSL2476*; *BPS_RS13300*), *folM* (*BPSS0039*; *BPS_RS18745*), and *folP* (*BPSL1357*; *BPS_RS07190*), and outer membrane protein Omp38 (*BPSS0879*; *BPS_RS23505*). Briefly, DNA sequences were aligned and annotated with the reference to observe for single-nucleotide polymorphisms (SNPs), insertion/deletions (indels), and gene duplication and amplification (GDA) events. Other mutations in the antibiotic targets, efflux pumps, and tetrahydrofolate biosynthetic pathways were explored on bacterial genomes. The variants involving alterations in amino acid were analyzed and compared with susceptible isolates.

### DNA sequencing.

The mutations involving *penA* sequences were verified by Sanger sequencing (SolGent, South Korea) using primer PenA1 and primer PenA2 pairs (see Table S1 in the supplemental material). The primers were designed using Primer-BLAST (https://ncbi.nlm.nih.gov/tools/primer-blast).

### Construction of *penA* deletion mutant and complemented strains.

A *penA* mutant and complemented B. pseudomallei using strain DR10212A were constructed based on pEXKm5 gene replacement as previously described (49, 50). Briefly, two fragment sequences containing a *penA*^DR10212A^ deletion and an insertion of *penA*^K96243^, respectively, were designed. These regions were flanked by homology sequences upstream and downstream of *penA* in DR10212A to allow for homologous recombination to integrate the desired mutations into the chromosome. A stop codon was included in the deletion fragment to generate a nonfunctional PenA. The *penA* deletion (pUC57*ΔpenA*^DR10212A^) and insertion (pCCI-4k::*penA*^K96243^) fragments were synthesized (Genscript, USA). The plasmid vectors were double digested with XhoI and EcoRI (TaKaRa, Japan). The correct fragment size was excised from the gel and purified using a QIAquick purification kit (Qiagen, Germany) and ligated to pEXKm5 using a Ligation Mighty Mix (TaKaRa, Japan). The plasmid was later transformed into E. coli DH5α, followed by RHO3. White kanamycin-resistant colonies were selected on LB agar with 35 μg/ml kanamycin containing 5-bromo-4-chloro-3-indolyl-β-d-galactopyranoside (X-Gal) (Promega, Italy). The colonies with constructed pEXKm5 were verified by correct fragment size by PCR using PenA-muta primers for the amplification of the internal region of *penA* (Table S1). Constructed pEXKm5-containing RHO3 colonies were later conjugated with B. pseudomallei on a nitrocellulose membrane on LB agar containing 400 μg/ml diaminopimelic acid (DAP). The merodiploid clones were selected and visualized as blue colonies on LB agar containing 1,000 μg/ml kanamycin and 5-bromo-4-chloro-3-indolyl-β-d-glucuronide (X-Gluc) (EMD Millipore, Switzerland). The correct fragment size was confirmed by PCR using primers described in Table S1. The clones upon *sacB*-mediated counterselection on sucrose containing yeast-extract tryptone agar were examined for kanamycin sensitivity. The *penA* sequences of kanamycin-sensitive mutants were verified by Sanger sequencing (SolGent, South Korea).

### Bacterial growth curve analysis.

Growth curve analysis was performed on B. pseudomallei isolates to assess the growth rate of antibiotic-resistant and laboratory-constructed mutant strains. Strain K96243 was used as the reference, and the initial isolate, strain DR50054A, was included for comparison with the relapse isolate, strain DR50054E. Briefly, one colony of B. pseudomallei was suspended in 3 ml LB broth and incubated at 37°C with shaking at 200 rpm overnight. The bacteria were then inoculated at a dilution of 1:100 into 5 ml of LB broth to obtain a bacterial concentration of approximately 1 × 10^6^ CFU/ml and incubated at 37°C with shaking at 200 rpm for 10 h. The optical density at 600 nm (OD_600_) value was recorded at 2-h intervals.

### RNA extraction and transcript quantification.

Two-step reverse transcription real-time PCR (RT-PCR) was used to compare *penA* transcriptions of B. pseudomallei strains grown in LB broth with and without CAZ, as previously described (27). Bacteria were harvested upon growth in LB broth at 37°C for 5 to 7 h upon reaching mid-log phase. For the antibiotic-induced condition, the bacteria were further incubated in LB medium with 32 μg/ml CAZ for an additional 2 h at 37°C with shaking at 200 rpm. RNA was extracted using TRIzol reagent (Invitrogen), followed by Turbo DNase treatment (Invitrogen). Genomic DNA removal was evaluated by the absence of the 16S rRNA gene using a SYBR green-based qPCR. The total RNA was converted to cDNA using iScript reverse transcription supermix (Bio-Rad) according to the manufacturer’s instruction. The quantitative assessment of the *penA* transcriptions was conducted in duplicate using iTaq universal SYBR green supermix (Bio-Rad) on a CFX96 Touch real-time PCR detection system (Bio-Rad). The RT-PCR conditions were as follows: initial denaturation at 95°C for 5 min, followed by 45 cycles of denaturation at 95°C for 15 s, and then annealing and extension at 54°C for 30 s. After amplification, melting curve analysis was conducted by increasing the annealing temperature by 0.1°C per step from 65°C to 95°C. The primer pair used was PenA4 (Table S1). The 16S rRNA gene was used as the housekeeping control for data normalization (51). The normalized expression levels were calculated using the formula: 2^−Δ^*^Cq^*, where the change in quantification cycle (Δ*C_q_*) = *C_q, penA_* − *C_q,_*_16S_. Student's *t* test was used to compare the differences in quantitative data between bacteria grown in LB broth with CAZ and bacteria grown in LB broth. The groups were considered statistically significant at a *P *of <0.05. All quantitative data are shown as the mean ± standard deviation (SD).

### β-Lactamase assay.

A β-lactamase assay was performed to quantify the β-lactamase activity in bacterial cultures as previously described (26). This is based on β-lactamase activity produced from bacteria via hydrolysis of a chromogenic cephalosporin, nitrocefin. Briefly, 20 μl of bacterial culture grown in LB medium to mid-log phase (OD_600_ = 0.5 to 0.6) was incubated with 0.2 ml of 0.5 mg/ml nitrocefin (Merck, USA) in 100 mM NaPO_4_ at pH 7 at 37°C in a 96-well plate. The absorbance was read at OD_486_ with a Tecan Sunrise microplate reader (Tecan, Switzerland). A mutant with the *penA* deletion, DR10212AΔ*penA*, was used as the negative control to eliminate the possible background activity of nitrocefin substrate. The Δ*A*_486_/min was calculated from the linear portion of the curve, and PenA activity units were calculated as (Δ*A*_486_/10^6^ cells × min) × 10^5^.

### Data availability.

The GenBank accession numbers for strain K96243 are NC_006350.1 and NC_006351.1. The accession numbers for the resistant and susceptible isolates are available under BioProject accession number PRJEB25606. The ENA and GenBank accession numbers for strains DR10212A, DR50054E, DR90049A, DR30013A, DR50054A, DR80110A, DR90031A, and DR90031E are SRR12710798, SRR12710797, SRR12710796, ERA1581689, ERA1581823, ERA1582124, ERA1582153, SRR12710795, respectively.

Sequences associated with specific PubMLST allele numbers can be retrieved from https://pubmlst.org/.

## Supplementary Material

Supplemental file 0
