# Supplementary material for: Antibiotic Susceptibility of Clinical Burkholderia pseudomallei Isolates in Northeast Thailand from 2015 to 2018 and the Genomic Characterization of β-Lactam-Resistant Isolates
Source: Antimicrob Agents Chemother. 2021 Apr 19;65(5):e02230-20. doi: 10.1128/AAC.02230-20 (PMC8092913; doi:10.1128/AAC.02230-20)
Supplement: Supplemental file 0 [file AAC.02230-20-s000S1.pdf]

Supplementary data

Table S1 Primers for *penA* used in this study

Primer	Primer sequence	Reference
**Sanger sequencing**		
PenA1-F	5'- ATTCGCACGCACTCCTGT	(1)
PenA1-R	5'-GTGAGCACGCGGATGAGC	
PenA2-F	5'- AATTGCGCGAACTCGAATC	This study
PenA2-R	5'- TCTTGTTGCCGAGCATCC	
**Gene expression**		
PenA4-F	5'- TGGATGCTCGGCAACAAGA	This study
PenA4-R	5'- TCGTTCGCCGTTCCGTAG	
16S-F	5'- GTGGGGAATTTTGGACAATG	(2)
16S-R	5'- CCGGGTATTAGCCAGAATGA	
**Mutagenesis**		
PenA-muta-F	5'- GATTCGCGCCCGTTCAATC	This study
PenA-muta-R	5'- CGGATTGCGCATCGTCGTAT	

Table S2 Mutations identified in clinical *Burkholderia pseudomallei* isolates with decreased antibiotic susceptibility in this study and previous studies.

Gene	Mutation	This study			Previous studies			References
		Isolate	MLST	Antibiotic and MIC (µg/ml)	Isolate	MLST	Antibiotic and MIC (µg/ml)	
**β-lactamases**								
Up-stream of *penA*	-78G>A	ND		ND	354e<br>MSHR99<br>MSHR1298<br>MSHR1300<br>MSHR1302<br>Bp1651	ST78<br>ST135<br>ST330<br>ST330<br>ST330<br>ST880	CAZ (6)<br>CAZ (16)<br>CAZ (≥256)<br>CAZ (≥256)<br>CAZ (≥256)<br>CAZ (>128)	(3-5)
	-49A>C	DR80110A	ST531	CAZ (16)	None		None	This study
penA	C69Y	ND		ND	7616y<br>7616w<br>3192<br>MSHR1226<br>MSHR1298<br>MSHR1300<br>MSHR1302<br>MSHR5654	NA<br>NA<br>NA<br>ST330<br>ST330<br>ST330<br>ST330<br>ST1040	CAZ (256)<br>CAZ (256)<br>CAZ (256)<br>CAZ (≥256)<br>CAZ (≥256)<br>CAZ (≥256)<br>CAZ (≥256)<br>CAZ (≥256)	(4, 6-7)
	P167S	DR10212A<br><br>DR30013A	ST10<br><br>ST10	CAZ (128)<br>MEM (8)<br>CAZ (64)	316c<br>P45	ST17<br>ST671	CAZ (64)<br>CAZ (64)	(8-9, This study)
	D240G	ND		ND	Bp1651	ST880	CAZ (>128)	(5)
	GDA	DR50054E	ST288	CAZ (64)	MSHR5654<br>MSHR8441<br>MSHR8442<br>5041c	ST1040<br>ST46<br>ST46<br>ST949	CAZ (≥256)<br>CAZ (12)<br>CAZ (2)<br>CAZ (32)	(7, 10, This study)

	S72F	ND		ND	392f<br>MSHR1655<br>Bp1651	NA<br>ST131<br>ST880	AMC (32)<br>AMC (32)<br>AMC (64)	(5, 8)
	T147A	DR50054A<br>DR50054E	ST288<br>ST288	AMC (8)<br>AMC (32)	Bp1651<br><br>MSHR0052<br>MSHR0664<br>MSHR0937	ST880<br><br>ST722<br>ST36<br>ST36	IPM (32)<br>AMC (64)<br>IPM (0.5), AMC (1.5-12)	(5, 11, This study)
**β-lactam target**								
PBP3	Deletion	ND		ND	415e<br>699d<br>1142b<br>3351<br>4236<br>4241	ST17<br>ST174<br>ST497<br>ST498<br>ST309<br>ST183	CAZ (>256)<br>CAZ (>256)<br>CAZ (>256)<br>CAZ (>256)<br>CAZ (>256)<br>CAZ (>256)	(1)
**AmrAB-OprA efflux pump**								
amrR	K13fs<br>E21D<br>G30D<br><br>G50E<br>V60_C63del<br>V71fs<br>L132P<br>A153_D156del<br>S166P<br>E190stop<br>V197del<br>A202_R207del<br>H92_S154del	ND<br>ND<br>ND<br><br>ND<br>ND<br>ND<br>ND<br>ND<br>ND<br>ND<br>DR10212A<br>DR90049A<br>DR90031E	 <br><br><br><br><br><br><br><br><br><br>ST10<br>ST207<br>ST207	ND<br>ND<br>ND<br><br>ND<br>ND<br>ND<br>ND<br>ND<br>ND<br>ND<br>SXT (4)<br>MEM (4)<br>MEM (4)	MSHR1300<br>MSHR0678<br>MSHR7587<br>MSHR7929<br>MSHR9872<br>MSHR6755<br>MSHR8481<br>MSHR5667<br>MSHR4083<br>MSHR9021<br>MSHR0052<br>None<br>None<br>None	ST330<br>ST114<br>ST437<br>ST437<br>ST1560<br>ST975<br>ST1378<br>ST252<br>ST36<br>ST132<br>ST722	MEM (4)<br>MEM (3)<br>MEM (3)<br>MEM (4)<br>MEM (6)<br>MEM (3)<br>MEM (6)<br>MEM (4)<br>MEM (6)<br>MEM (3)<br>MEM (8)<br>None<br>None<br>None	(7, 11-12, This study)

**BpeAB-OprB efflux pump**								
bpeR	L85fs<br>D176A	ND<br>ND		ND<br>ND	MSHR0800<br>MSHR0937	ST114<br>ST36	MEM (6)<br>MEM (6)	(11-12)
**BpeEF-OprC efflux pump**								
oprC	W154fs	DR90049A	ST207	MEM (4)	None		None	This study
bpeT	Truncated*<br>S311R<br><br>T314fs 	ND<br><br>ND<br><br>ND		ND<br><br>ND<br><br>ND	354e<br><br>MSHR1300<br><br>MSHR5654	ST78<br><br>ST330<br><br>ST1040	MEM (6)<br>SXT (3)<br>MEM (4)<br>SXT (3)<br>SXT (≥32)	(3, 7, 11-13)
bpeS	P29S<br><br><br>V40I<br>R247L<br>A311fs	ND<br><br><br>ND<br>ND<br>DR90049A	 <br><br><br><br>ST207	ND<br><br><br>ND<br>ND<br>MEM (4)	1374a<br>5041a<br>Bp1651<br>MSHR8441<br>MSHR8442<br>None	NA<br>NA<br>ST880<br>ST46<br>ST46 	SXT (4)<br>SXT (4)<br>SXT (>32)<br>SXT (≥32)<br>SXT (≥32)<br>None	(5, 13-14, This study)
**Outer membrane porin**								
Omp38		ND			None		None	
**Tetra-hydrofolate biosynthesis pathway**								
folM	R20_A22ins<br><br>R20fs 	ND<br><br>ND 		ND<br><br>ND 	MSHR8441<br>MSHR8442<br>5041a<br>354e	ST46<br>ST46<br>NA<br>ST78	SXT (≥32)<br>SXT (≥32)<br>SXT (4)<br>SXT (3)	(3, 7, 14)

ND, not detected; NA, not available; * truncated *bpeT* carboxy terminus due to 800-kb inversion in chromosome 2; Values reported for AMC and SXT indicated amoxicillin and trimethoprim concentration, respectively; Putative mutations found in this study were colored blue.

Table S3 Amplified coding gene regions in chromosome 2 of *Burkholderia pseudomallei* strain DR50054E

Old locus tag	New NCBI locus tag	Location (CDS)		Gene product	Average read coverage
		Start	End		
BPSS0944	*BPS_RS23855*	1245541	1246452	LysR family transcriptional regulator	335
NA	*BPS_RS23860*	1246731	1247027	Hypothetical protein	657
BPSS0945	*BPS_RS23865*	1247073	1248020	M23 family metallopeptidase	218
BPSS0946	*BPS_RS23870*	1248195	1249082	Class A β-lactamase (PenA)	236
NA	*BPS_RS23875*	1249259	1249774	Hypothetical protein	181
BPSS0948	*BPS_RS23880*	1249773	1250645	LysR family transcriptional regulator	473
BPSS0949	*BPS_RS23885*	1250756	1251091	Hypothetical protein	391
BPSS0950	*BPS_RS23890*	1251939	1252727	ABC transporter ATP-binding protein	372
BPSS0951	*BPS_RS23895*	1252715	1253584	ABC transporter ATP-binding protein	174
BPSS0952	*BPS_RS23900*	1253586	1254527	ABC transporter permease	226
BPSS0953	*BPS_RS23905*	1254570	1255640	ABC transporter permease	385
BPSS0954	*BPS_RS23910*	1255661	1257262	ABC transporter substrate-binding protein	711
BPSS0955	*BPS_RS23915*	1257411	1257980	D-alanyl-D-alanine dipeptidase	325
BPSS0956	*BPS_RS23920*	1258000	1258890	MurR/RpiR family transcriptional regulator	361

NA	*BPS_RS23925*	1259003	1259245	Hypothetical protein	19
NA	*BPS_RS23930*	1259374	1259655	Hypothetical protein	656
BPSS0957	*BPS_RS23935*	1259881	1261029	Hypothetical protein	255
BPSS0958	*BPS_RS23940*	1261382	1263367	type VI secretion system tip protein VgrG	788
BPSS0959	*BPS_RS23945*	1263459	1263899	DUF1795 domain-containing protein	644
BPSS0960	*BPS_RS23950*	1263951	1268576	Rhs-related membrane protein	465

NA, not available

Table S4 Antibiotic susceptibilities for primary episode of clinical *Burkholderia pseudomallei* isolates in different countries. The data were obtained from previous studies and this study.

Country	Year	No. of isolates tested	Antibiotic susceptibility (%)					References
			CAZ	MEM	IPM	AMC	SXT	
Australia[1]	2009-2012	234	100	100	ND	ND	99.1	(15)
Cambodia[2]	2007	39	100	ND	100	82.1	100[1]	(16)
Cambodia[1]	2007-2010	52	100	100	ND	100	100	(17)
Cambodia[1]	2006-2012	620	ND	ND	ND	ND	100	(18)
Laos[1]	2003-2012	149	ND	ND	ND	ND	99.2	(18)
Malaysia[1]	2001-2009	170	99.4	100	99.4	ND	90	(19)
Malaysia[2]	2005-2010	228	97.6	ND	90.2	82	63	(20)
Malaysia[1]	2001-2013	138	99.3	100	100	100	61.7	(21)
Thailand[2]	2000-2004	WHONET data	>98.5	98	98.5	>95.0	<53	(22)
Thailand[1,2]	1987-2007	4,021	99.9	100	100	99.9	ND	(23)
Thailand[1]	1992-2003	3038	ND	ND	ND	ND	99.7	(24)
Thailand[2]	2010-2011	85	100	ND	ND	99	ND	(25)
Thailand[2,3]	2015-2018	1,304	99.8	99.8	100	99.9	ND	This study
Vietnam[1]	2012-2017	312	100	ND	100	100	89.1	(26)

[1] E-test; [2] Disk diffusion; [3] Broth microdilution; NARST, National Antimicrobial Resistance Surveillance; IPM, imipenem; MEM, meropenem; CAZ, ceftazidime; SXT, trimetoprim-sulfamethoxazole; AMC, amoxicillin-clavulanic acid; ND, not done

References

1. Chantratita N, Rholl DA, Sim B, Wuthiekanun V, Limmathurotsakul D, Amornchai P, Thanwisai A, Chua HH, Ooi WF, Holden MT, Day NP, Tan P, Schweizer HP, Peacock SJ. 2011. Antimicrobial resistance to ceftazidime involving loss of penicillin-binding protein 3 in *Burkholderia pseudomallei*. Proc Natl Acad Sci U S A 108:17165-70.
2. Paksanont S, Sintiprungrat K, Yimthin T, Pumirat P, Peacock SJ, Chantratita N. 2018. Effect of temperature on *Burkholderia pseudomallei* growth, proteomic changes, motility and resistance to stress environments. Sci Rep 8:9167.
3. Hayden HS, Lim R, Brittnacher MJ, Sims EH, Ramage ER, Fong C, Wu Z, Crist E, Chang J, Zhou Y, Radey M, Rohmer L, Haugen E, Gillett W, Wuthiekanun V, Peacock SJ, Kaul R, Miller SI, Manoil C, Jacobs MA. 2012. Evolution of *Burkholderia pseudomallei* in recurrent melioidosis. PLoS One 7:e36507.
4. Sarovich DS, Price EP, Von Schulze AT, Cook JM, Mayo M, Watson LM, Richardson L, Seymour ML, Tuanyok A, Engelthaler DM, Pearson T, Peacock SJ, Currie BJ, Keim P, Wagner DM. 2012a. Characterization of ceftazidime resistance mechanisms in clinical isolates of *Burkholderia pseudomallei* from Australia. PLoS One 7:e30789.
5. Bugrysheva JV, Sue D, Gee JE, Elrod MG, Hoffmaster AR, Randall LB, Chirakul S, Tuanyok A, Schweizer HP, Weigel LM. 2017. Antibiotic Resistance Markers in *Burkholderia pseudomallei* Strain Bp1651 Identified by Genome Sequence Analysis. Antimicrob Agents Chemother 61.
6. Sam IC, See KH, Puthucheary SD. 2009. Variations in ceftazidime and amoxicillin-clavulanate susceptibilities within a clonal infection of *Burkholderia pseudomallei*.

J Clin Microbiol 47:1556-8.

7. Viberg LT, Sarovich DS, Kidd TJ, Geake JB, Bell SC, Currie BJ, Price EP. 2017. Within-Host Evolution of *Burkholderia pseudomallei* during Chronic Infection of Seven Australasian Cystic Fibrosis Patients. mBio 8.

8. Tribuddharat C, Moore RA, Baker P, Woods DE. 2003. *Burkholderia pseudomallei* class a beta-lactamase mutations that confer selective resistance against ceftazidime or clavulanic acid inhibition. Antimicrob Agents Chemother 47:2082-7.

9. Sarovich DS, Price EP, Limmathurotsakul D, Cook JM, Von Schulze AT, Wolken SR, Keim P, Peacock SJ, Pearson T. 2012b. Development of ceftazidime resistance in an acute *Burkholderia pseudomallei* infection. Infect Drug Resist 5:129-32.

10. Chirakul S, Somprasong N, Norris MH, Wuthiekanun V, Chantratita N, Tuanyok A, Schweizer HP. 2019. *Burkholderia pseudomallei* acquired ceftazidime resistance due to gene duplication and amplification. Int J Antimicrob Agents 53:582-588.

11. Sarovich DS, Webb JR, Pitman MC, Viberg LT, Mayo M, Baird RW, Robson JM, Currie BJ, Price EP. 2018. Raising the Stakes: Loss of Efflux Pump Regulation Decreases Meropenem Susceptibility in *Burkholderia pseudomallei*. Clin Infect Dis 67:243-250.

12. Webb JR, Price EP, Somprasong N, Schweizer HP, Baird RW, Currie BJ, Sarovich DS. 2018. Development and validation of a triplex quantitative real-time PCR assay

to detect efflux pump-mediated antibiotic resistance in Burkholderia pseudomallei. Future Microbiol 13:1403-1418.

13. Price EP, Viberg LT, Kidd TJ, Bell SC, Currie BJ, Sarovich DS. 2018. Transcriptomic analysis of longitudinal Burkholderia pseudomallei infecting the cystic fibrosis lung. Microb Genom 4.

14. Podnecky NL, Rhodes KA, Mima T, Drew HR, Chirakul S, Wuthiekanun V, Schupp JM, Sarovich DS, Currie BJ, Keim P, Schweizer HP. 2017. Mechanisms of Resistance to Folate Pathway Inhibitors in Burkholderia pseudomallei: Deviation from the Norm. mBio 8.

15. Crowe A, McMahon N, Currie BJ, Baird RW. 2014. Current antimicrobial susceptibility of first-episode melioidosis *Burkholderia pseudomallei* isolates from the Northern Territory, Australia. Int J Antimicrob Agents 44:160-2.

16. Rammaert B, Beaute J, Borand L, Hem S, Buchy P, Goyet S, Overtoom R, Angebault C, Te V, Try PL, Mayaud C, Vong S, Guillard B. 2011. Pulmonary melioidosis in Cambodia: a prospective study. BMC Infect Dis 11:126.

17. Vlieghe E, Kruy L, De Smet B, Kham C, Veng CH, Phe T, Koole O, Thai S, Lynen L, Jacobs J. 2011. Melioidosis, phnom penh, Cambodia. Emerg Infect Dis 17:1289-92.

18. Dance D. 2014. Treatment and prophylaxis of melioidosis. Int J Antimicrob Agents 43:310-8.

19. Ahmad N, Hashim R, Mohd Noor A. 2013. The In Vitro Antibiotic Susceptibility of Malaysian Isolates of *Burkholderia pseudomallei*. Int J Microbiol 2013:121845.

20. Hassan MR, Vijayalakshmi N, Pani SP, Peng NP, Mehenderkar R, Voralu K, Michael E. 2014. Antimicrobial susceptibility patterns of *Burkholderia pseudomallei* among melioidosis cases in Kedah, Malaysia. Southeast Asian J Trop Med Public Health 45:680-8.

21. Zueter A, Yean CY, Abumarzouq M, Rahman ZA, Deris ZZ, Harun A. 2016. The epidemiology and clinical spectrum of melioidosis in a teaching hospital in a North-Eastern state of Malaysia: a fifteen-year review. BMC Infect Dis 16:333.

22. Paveenkittiporn W, Apisarnthanarak A, Dejsirilert S, Trakulsomboon S, Thongmali O, Sawanpanyalert P, Aswapokee N. 2009. Five-year surveillance for *Burkholderia pseudomallei* in Thailand from 2000 to 2004: prevalence and antimicrobial susceptibility. J Med Assoc Thai 92 Suppl 4:S46-52.

23. Wuthiekanun V, Amornchai P, Saiprom N, Chantratita N, Chierakul W, Koh GC, Chaowagul W, Day NP, Limmathurotsakul D, Peacock SJ. 2011. Survey of antimicrobial resistance in clinical *Burkholderia pseudomallei* isolates over two decades in Northeast Thailand. Antimicrob Agents Chemother 55:5388-91.

24. Saiprom N, Amornchai P, Wuthiekanun V, Day NP, Limmathurotsakul D, Peacock SJ, Chantratita N. 2015. Trimethoprim/sulfamethoxazole resistance in clinical isolates of Burkholderia pseudomallei from Thailand. Int J Antimicrob Agents 45:557-9.

25. Panya M, Thirat S, Wanram S, Panomket P, Nilsakul J. 2016. Prevalence of bla(PenA) and bla(OXA) in *Burkholderia pseudomallei* Isolated from Patients at

Sappasitthiprasong Hospital and Their Susceptibility to Ceftazidime and Carbapenems. J Med Assoc Thai, 99 Suppl 1, S12-16.

26. Nhung PH, Van VH, Anh NQ, Phuong DM. 2019. Antimicrobial susceptibility of *Burkholderia pseudomallei* isolates in Northern Vietnam. J Glob Antimicrob Resist 18:34-36.
